# Urine steroid metabolomics for the differential diagnosis of adrenal incidentalomas in the EURINE-ACT study: a prospective test validation study

**DOI:** 10.1016/S2213-8587(20)30218-7

**Published:** 2020-09

**Authors:** Irina Bancos, Angela E Taylor, Vasileios Chortis, Alice J Sitch, Carl Jenkinson, Caroline J Davidge-Pitts, Katharina Lang, Stylianos Tsagarakis, Magdalena Macech, Anna Riester, Timo Deutschbein, Ivana D Pupovac, Tina Kienitz, Alessandro Prete, Thomas G Papathomas, Lorna C Gilligan, Cristian Bancos, Giuseppe Reimondo, Magalie Haissaguerre, Ljiljana Marina, Marianne A Grytaas, Ahmed Sajwani, Katharina Langton, Hannah E Ivison, Cedric H L Shackleton, Dana Erickson, Miriam Asia, Sotiria Palimeri, Agnieszka Kondracka, Ariadni Spyroglou, Cristina L Ronchi, Bojana Simunov, Danae A Delivanis, Robert P Sutcliffe, Ioanna Tsirou, Tomasz Bednarczuk, Martin Reincke, Stephanie Burger-Stritt, Richard A Feelders, Letizia Canu, Harm R Haak, Graeme Eisenhofer, M Conall Dennedy, Grethe A Ueland, Miomira Ivovic, Antoine Tabarin, Massimo Terzolo, Marcus Quinkler, Darko Kastelan, Martin Fassnacht, Felix Beuschlein, Urszula Ambroziak, Dimitra A Vassiliadi, Michael W O'Reilly, William F Young, Michael Biehl, Jonathan J Deeks, Wiebke Arlt, ENSAT EURINE-ACT Investigators, Stephan Glöckner, Richard O. Sinnott, Anthony Stell, Maria C. Fragoso, Darko Kastelan, Ivana D. Pupovac, Bojana Simunov, Sarah Cazenave, Magalie Haissaguerre, Antoine Tabarin, Jérôme Bertherat, Rossella Libé, Tina Kienitz, Marcus Quinkler, Katharina Langton, Graeme Eisenhofer, Felix Beuschlein, Christina Brugger, Martin Reincke, Anna Riester, Ariadni Spyroglou, Stephanie Burger-Stritt, Timo Deutschbein, Martin Fassnacht, Stefanie Hahner, Matthias Kroiss, Cristina L. Ronchi, Sotiria Palimeri, Stylianos Tsagarakis, Ioanna Tsirou, Dimitra A. Vassiliadi, Vittoria Basile, Elisa Ingargiola, Giuseppe Reimondo, Massimo Terzolo, Letizia Canu, Massimo Mannelli, Hester Ettaieb, Harm R. Haak, Thomas M. Kerkhofs, Michael Biehl, Richard A. Feelders, Johannes Hofland, Leo J. Hofland, Marianne A. Grytaas, Eystein S. Husebye, Grethe A. Ueland, Urszula Ambroziak, Tomasz Bednarczuk, Agnieszka Kondracka, Magdalena Macech, Malgorzata Zawierucha, Isabel Paiva, M. Conall Dennedy, Ahmed Sajwani, Mark Sherlock, Rachel K. Crowley, Miomira Ivovic, Ljiljana Marina, Jonathan J. Deeks, Alice J. Sitch, Wiebke Arlt, Irina Bancos, Vasileios Chortis, Lorna C. Giligan, Beverly A. Hughes, Katharina Lang, Hannah E. Ivison, Carl Jenkinson, Konstantinos Manolopoulos, Donna M. O'Neil, Michael W. O'Reilly, Thomas G. Papathomas, Alessandro Prete, Cedric H.L. Shackleton, Angela E. Taylor, Miriam Asia, Robert P. Sutcliffe, Peter Guest, Kassiani Skordilis, Cristian Bancos, Alice Chang, Caroline J. Davidge-Pitts, Danae A. Delivanis, Dana Erickson, Neena Natt, Todd B. Nippoldt, Melinda Thomas, William F. Young Jr.

**Affiliations:** a Institute of Metabolism and Systems Research, University of Birmingham, Birmingham, UK; b Institute of Applied Health Research, University of Birmingham, Birmingham, UK; c Division of Endocrinology, Diabetes, Metabolism and Nutrition, Mayo Clinic, Rochester, MN, USA; d Centre for Endocrinology, Diabetes and Metabolism, Birmingham Health Partners, Birmingham, UK; e Department of Endocrinology, Queen Elizabeth Hospital, University Hospitals Birmingham NHS Foundation Trust, Birmingham, UK; f Department of Hepato-Pancreato-Biliary and Liver Transplant Surgery, Queen Elizabeth Hospital, University Hospitals Birmingham NHS Foundation Trust, Birmingham, UK; g NIHR Birmingham Biomedical Research Centre, University Hospitals Birmingham NHS Foundation Trust and University of Birmingham, Birmingham, UK; h Department of Endocrinology, Diabetes and Metabolism, Evangelismos Hospital, Athens, Greece; i Department of Internal Medicine and Endocrinology, Medical University of Warsaw, Warsaw, Poland; j Medizinische Klinik and Poliklinik IV, Klinikum der Universität, Ludwig-Maximilians-Universität München, Munich, Germany; k Division of Endocrinology and Diabetes, Department of Internal Medicine I, University Hospital Würzburg, University of Würzburg, Würzburg, Germany; l Comprehensive Cancer Center Mainfranken, University Hospital Würzburg, University of Würzburg, Würzburg, Germany; m Central Laboratory, University Hospital Würzburg, University of Würzburg, Würzburg, Germany; n Department of Endocrinology, University Hospital Centre Zagreb, Zagreb, Croatia; o Endocrinology in Charlottenburg, Berlin, Germany; p Department of Clinical and Biological Sciences, San Luigi Hospital, University of Turin, Turin, Italy; q Department of Endocrinology, Hôpital Haut Lévêque, CHU de Bordeaux, Pessac, France; r Department for Obesity, Reproductive and Metabolic Disorders, Clinic for Endocrinology, Diabetes and Metabolic Diseases, Clinical Centre of Serbia, Faculty of Medicine, University of Belgrade, Belgrade, Serbia; s Department of Clinical Science, University of Bergen, Bergen, Norway; t Department of Medicine, Haukeland University Hospital, Bergen, Norway; u School of Medicine, National University of Ireland Galway, Galway, Ireland; v Institute of Clinical Chemistry and Laboratory Medicine, University Hospital Carl Gustav Carus, Technical University, Dresden, Germany; w UCSF Benioff Children's Hospital Oakland Research Institute, Oakland, CA, USA; x Department of Internal Medicine, Division of Endocrinology, Erasmus University Medical Centre, Rotterdam, Netherlands; y Department of Experimental and Clinical Biomedical Sciences, University of Florence, Florence, Italy; z Department of Internal Medicine, Maxima Medisch Centrum, Eindhoven, Netherlands; aa Department of Health Services Research and CAPHRI School for Public Health and Primary Care, Maastricht University, Maastricht, Netherlands; ab Klinik für Endokrinologie, Diabetologie und Klinische Ernährung, Universitätsspital Zürich, Zurich, Switzerland; ac Bernoulli Institute for Mathematics, Computer Science and Artificial Intelligence, University of Groningen, Groningen, Netherlands

## Abstract

**Background:**

Cross-sectional imaging regularly results in incidental discovery of adrenal tumours, requiring exclusion of adrenocortical carcinoma (ACC). However, differentiation is hampered by poor specificity of imaging characteristics. We aimed to validate a urine steroid metabolomics approach, using steroid profiling as the diagnostic basis for ACC.

**Methods:**

We did a prospective multicentre study in adult participants (age ≥18 years) with newly diagnosed adrenal masses. We assessed the accuracy of diagnostic imaging strategies based on maximum tumour diameter (≥4 cm *vs* <4 cm), imaging characteristics (positive *vs* negative), and urine steroid metabolomics (low, medium, or high risk of ACC), separately and in combination, using a reference standard of histopathology and follow-up investigations. With respect to imaging characteristics, we also assessed the diagnostic utility of increasing the unenhanced CT tumour attenuation threshold from the recommended 10 Hounsfield units (HU) to 20 HU.

**Findings:**

Of 2169 participants recruited between Jan 17, 2011, and July 15, 2016, we included 2017 from 14 specialist centres in 11 countries in the final analysis. 98 (4·9%) had histopathologically or clinically and biochemically confirmed ACC. Tumours with diameters of 4 cm or larger were identified in 488 participants (24·2%), including 96 of the 98 with ACC (positive predictive value [PPV] 19·7%, 95% CI 16·2–23·5). For imaging characteristics, increasing the unenhanced CT tumour attenuation threshold to 20 HU from the recommended 10 HU increased specificity for ACC (80·0% [95% CI 77·9–82·0] *vs* 64·0% [61·4–66.4]) while maintaining sensitivity (99·0% [94·4–100·0] *vs* 100·0% [96·3–100·0]; PPV 19·7%, 16·3–23·5). A urine steroid metabolomics result indicating high risk of ACC had a PPV of 34·6% (95% CI 28·6–41·0). When the three tests were combined, in the order of tumour diameter, positive imaging characteristics, and urine steroid metabolomics, 106 (5·3%) participants had the result maximum tumour diameter of 4 cm or larger, positive imaging characteristics (with the 20 HU cutoff), and urine steroid metabolomics indicating high risk of ACC, for which the PPV was 76·4% (95% CI 67·2–84·1). 70 (3·5%) were classified as being at moderate risk of ACC and 1841 (91·3%) at low risk (negative predictive value 99·7%, 99·4–100·0).

**Interpretation:**

An unenhanced CT tumour attenuation cutoff of 20 HU should replace that of 10 HU for exclusion of ACC. A triple test strategy of tumour diameter, imaging characteristics, and urine steroid metabolomics improves detection of ACC, which could shorten time to surgery for patients with ACC and help to avoid unnecessary surgery in patients with benign tumours.

**Funding:**

European Commission, UK Medical Research Council, Wellcome Trust, and UK National Institute for Health Research, US National Institutes of Health, the Claire Khan Trust Fund at University Hospitals Birmingham Charities, and the Mayo Clinic Foundation for Medical Education and Research.

## Introduction

Adrenal masses are discovered incidentally in about 5% of cross-sectional imaging examinations.^1^, ^2^ The prevalence of these so-called incidentalomas increases with age and is estimated to be about 3% among people aged 40 years and 10% among those aged 70 years.^3^ Because of widespread use of CT and MRI, the number of adrenal incidentalomas identified is increasing worldwide. In the USA, more than 100 million CT and MRI examinations are done per year,^4^ which could potentially reveal around 5 million adrenal masses. Diagnostic assessment and therapeutic management of incidentalomas is a challenging health-care issue.^5^, ^6^

**Research in context**

**Evidence before this study**

The discovery of an adrenal mass requires further assessment to determine whether the tumour is a malignant adrenocortical carcinoma (ACC) or a benign adrenocortical adenoma (ACA). Imaging tests are the mainstay in detection of ACC, but the available methods have poor diagnostic accuracy. In 2016 we did a systematic review and meta-analysis of the accuracy of imaging modalities. We searched MEDLINE, Embase, the Cochrane Central Register of Controlled Trials, the Science Citation Index, the Conference Proceedings Citation Index, and Zetoc for reports published from Jan 1, 1990, to Aug 31, 2015, to identify all studies that had assessed the diagnostic accuracy of imaging methods employed in the differential diagnosis of adrenal masses (CT, MRI, and ^18^F-fluorodeoxyglucose [^18^F-FDG] PET) in comparison with histology or imaging-based follow-up as the reference standard. The search terms used for these database searches were reported in that study. Based on data from only two eligible studies involving 102 participants, an unenhanced CT tumour attenuation value of more than 10 Hounsfield units (HU) was shown to have high sensitivity (100%) but poor specificity (72%) for the detection of ACC. Studies assessing ^18^F-FDG PET, MRI with chemical shift analysis, and CT with contrast washout had even smaller sample sizes (n=25–75) and generated sensitivity and specificity estimates with very wide CIs. In 2019 we updated our previous search with a PubMed search using the same search terms and covering the dates Sept 1, 2015–Jan 31, 2019. We found no studies that altered the conclusions of the 2016 systematic review and meta-analysis, nor any newly published meta-analyses addressing the same question. We did a retrospective proof-of-principle study in 45 participants with ACC and 102 with ACA, in which we showed that urine steroid metabolomics (ie, mass spectrometry-based steroid metabolite profiling of 24 h urine samples combined with machine-learning-based data analysis) could detect ACC with high sensitivity and specificity. Although similar diagnostic accuracies were reported in several other retrospective studies, we did not identify any previous prospective test validation studies.

**Added value of this study**

In this prospective, multicentre, test validation study, we assessed the diagnostic accuracy of urine steroid metabolomics for the detection of ACC in patients with newly diagnosed adrenal masses compared with routinely employed imaging strategies (measurement of tumour size and assessment of imaging characteristics). As a single test, urine steroid metabolomics had a higher positive predictive value (PPV) than either imaging test (34·6**%** [95% CI 28·6–41·0] *vs* 19·7% [16·2–23·5] for tumour size and 19·7% [16·3–23·5] for imaging characteristics). Additionally, in a comparison with the standarad cutoff of 10 HU for unenhanced CT tumour attenuation, a cutoff of 20 HU increased specificity from 64% to 80% while sensitivity remained similar. The best diagnostic performance was provided by the combination of tumour diameter greater than 4 cm, unenhanced CT tumour attenuation greater than 20 HU, and urine steroid metabolomics indicating a high risk of ACC, which provided a PPV of 76·4% and a negative predictive value of 99·7% for ACC.

**Implications of all the available evidence**

This study prospectively validated the diagnostic accuracy of urine steroid metabolomics seen in retrospective studies. Use of radiation-free, non-invasive urine steroid metabolomics has a higher PPV than two standard imaging tests, and best performance was seen with the combination of all three tests. Implementation of urine steroid metabolomics in the routine diagnostic assessment of newly discovered adrenal masses could reduce the number of imaging procedures required to diagnose ACC and avoid unnecessary surgery of benign adrenal tumours, potentially yielding beneficial effects with respect to patient burden and health-care costs.

The discovery of an adrenal mass prompts two major questions to be addressed by the diagnostic investigations. The first question is whether the tumour causes adrenal hormone excess, which requires exclusion of pheochromocytoma, Cushing's syndrome, and primary aldosteronism via biochemical testing.^7^, ^8^, ^9^ The second question is whether the adrenal mass is an adrenocortical carcinoma (ACC). These tumours account for 2–12% of adrenal incidentalomas.^10^, ^11^, ^12^, ^13^ Prognosis of ACC is poor and cure is achievable only with early detection and surgery.^14^ Multimodal and repeated imaging is routinely used to detect and exclude ACC, despite an insufficient evidence base to support this approach due to small studies in heterogeneous populations and with poor reference standards.^15^ Based on available data, an unenhanced CT tumour attenuation of less than 10 Hounsfield units (HU) is thought to be indicative of a lipid-rich mass, which is a common characteristic of benign adrenal tumours.^15^ European guidelines, therefore, recommend that unenhanced CT should be the primary imaging test to exclude ACC.^16^ However, the 10 HU cutoff has poor specificity for malignancy because of the existence of many lipid-poor benign adrenal masses.^11^, ^15^, ^17^ Patients therefore frequently undergo repeated imaging by multiple modalities and the unnecessary surgical removal of adrenal masses that are revealed postoperatively to be benign.^10^, ^11^

Urine steroid metabolomics is the combination of mass spectrometry-based urinary steroid metabolite profiling and machine-learning-based data analysis. In a retrospective proof-of-principle study, we showed that this approach was highly promising for the detection of ACC, with high sensitivity (90%) and specificity (88%).^18^ Similar findings were reported in several subsequent retrospective studies.^19^, ^20^, ^21^

To validate the use of urine steroid metabolomic testing in this context, we did the prospective, multicentre Evaluation of Urine Steroid Metabolomics in the Differential Diagnosis of Adrenocortical Tumours (EURINE-ACT) study in adult participants with newly diagnosed adrenal masses. We investigated the diagnostic accuracy of urine steroid metabolomics alone and in combination with standard imaging protocols, and compared our findings with histopathology and clinical and imaging follow-up investigations as the reference standard.

## Methods

### Study design and participants

EURINE-ACT was a prospective test validation study performed according to the STARD guidelines for studies of diagnostic accuracy (appendix pp 36–37) and done in adult participants (age ≥18 years) with a newly identified adrenal mass of more than 1 cm diameter. Exclusion criteria were biochemical evidence of pheochromocytoma (appendix p 5), pregnancy, lactation, and current or recent (<6 months) intake of drugs known to alter steroid synthesis or metabolism. Patients with an adrenal mass discovered during imaging for cancer staging or monitoring were also not eligible.

Participants were recruited through specialist centres participating in the European Network for the Study of Adrenal Tumours ([ENSAT]; appendix p 4). The study was advertised to all ENSAT members, and 21 centres in 14 countries (Brazil, Croatia, France, Germany, Greece, Ireland, Italy, the Netherlands, Norway, Poland, Portugal, Serbia, the UK, and the USA) agreed to participate and initiated enrolment. We asked centres to recruit prospectively consecutive eligible individuals willing to participate (ie, non-selective recruitment), from Jan 17, 2011, to July 15, 2016.

All participating centres obtained local ethics approval for recording of pseudonymised and standardised data in the ENSAT registry relevant to the study (demographic characteristics, method of tumour identification, tumour diameter and imaging characteristics, endocrine testing results, clinical and radiological follow-up data, surgery details, and histopathology data) and for collection and use of participant-related biomaterial (appendix pp 4–5). All participants provided written informed consent before inclusion.

### Reference standard

The reference standard for ACC was based on histopathology or, alternatively, the presence of a large adrenal tumour and mixed steroid excess typical of ACC, with no other feasible alternative diagnosis. Adrenal masses were classified as benign based on histopathology after surgical removal, or, in those not removed, by lack of growth on imaging after 6 months or clinical folllow-up of at least 12 months (appendix p 23).

### Imaging assessments

Diagnostic investigation for adrenal tumours by imaging was done as part of routine care at the clinical centres in accordance with their standard guidelines. Local centres made decisions about the need for adrenalectomy (and in rare cases adrenal biopsy) based on imaging, without any access to urine steroid metabolomics data. The imaging index tests we recorded were maximum tumour diameter at the time of discovery and imaging characteristics. Tumour diameter of 4 cm or greater indicated suspicion of ACC.^10^

Imaging characteristics regarded as suspicious of ACC^11^, ^15^ were as follows (in order of ranking^15^): unenhanced CT tumour tissue attenuation (<10 HU, 10–20 HU, or >20 HU in homogeneous tumours or heterogeneous tumours precluding HU measurement); MRI chemical shift analysis with no loss of signal intensity in the tumour area on out-of-phase images; ^18^F-fluorodeoxyglucose (^18^F-FDG) PET with a tumour standardised uptake value higher than that in the liver; follow-up imaging showing an increase of the maximum diameter of at least 20% 6 months or more after the index scan; and CT contrast washout assessment showing absolute contrast washout from less than 60% of the tumour area. If participants underwent multiple imaging modalities, a positive or negative result for ACC was based on the highest ranking imaging characteristic reported.

### Urine steroid metabolomics testing

Enrolled participants collected a 24 h urine sample that was used for multisteroid profiling by liquid chromatography–tandem mass spectrometry (LC–MS/MS), with quantification of 15 urinary steroid metabolites (appendix p 19) and application of a machine-learning algorithm.

The algorithm was developed by applying generalised matrix learning vector quantisation^18^ to steroid excretion data from a retrospective cohort of 139 patients with adrenal masses (40 ACC and 99 adrenocortical adenoma [ACA]) measured retrospectively by use of the LC–MS/MS method used in this study (appendix pp 7–9). Based on the distances of the entirety of the steroid metabolome of ACC and ACA prototypes, the generalised matrix learning vector quantisation classifier provides a test outcome score. The corresponding thresholds were selected to ensure the post-test probability of ACC was greater than 65% in the high-risk group and less than 10% in the low-risk group, giving a moderate risk range of 10–65% (appendix p 9).

Urine steroid metabolomics analyses were done after all routine tests in the study centres were completed, but without access to the reference standard findings or other diagnostic information. This timing also prevented urine steroid metabolomics results being communicated to the study centres and affecting the normal diagnostic process.

### Statistical analysis

We aimed to include 2000 participants and expected an ACC rate of 5%, based on the results of our proof-of-principle study (appendix p 4), and loss to follow-up of 10%.^18^ We calculated that observing 100 ACC cases would provide 95% sensitivity with a 95% CI range of less than 10% and more than 99% power to detect a difference of 3% in specificity (87% *vs* 90%) between standard imaging protocols and urine steroid metabolomics at the 5% significance level.

Characteristics of participants are reported for each type of tumour assessed (ACC, other malignant tumours, ACA, and other benign tumours), with data presented as median (IQR) for categorical results or number (%) for continuous data. For each diagnostic test (maximum tumour diameter [≥4 cm *vs* <4], imaging characteristics [positive *vs* negative], or urine steroid metabolomics [low, medium, or high risk of ACC]), we computed the percentage of ACC cases with each test result (giving sensitivity for a positive result in the binary tests); the percentage of non-ACC cases with each test result (giving specificity for a negative result in the binary tests); and the likelihood ratio for each test result. Additionally, we assessed the tests in combinations of two (tumour diameter plus imaging characteristics, tumour diameter plus urine steroid metabolomics, and imaging characteristics plus urine steroid metabolomics) and as a triple test strategy (tumour diameter, followed by imaging characteristics followed by urine steroid metabolomics). We also calculated the proportions of participants with ACC who had each test result to estimate the probability of ACC (ie, positive predictive value [PPV] for positive results and 1 minus negative predictive value [NPV] for negative test results). All results are reported with 95% CIs, computed by use of the exact binomial method for proportions and the Wald-based methods for likelihood ratios. Finally, we investigated the diagnostic utility of increasing the unenhanced CT tumour attenuation threshold from the recommended 10 HU to 20 HU.

For urine steroid metabolomics, we calculated the area under the receiver operating characteristic curve (AUROC) with 95% CIs. A sensitivity analysis was done that excluded participants with ACC who had mixed or aberrant steroid excess, clinical presentation of large adrenal mass with extra-adrenal metastases, or bilateral macronodular adrenal hyperplasia and isolated cortisol excess identifying presumed benign tumours.

All statistical analyses were done with Stata version 16 and graphs were created in R.

### Role of the funding source

The funders of the study had no role in study design, data collection, data analysis, data interpretation, or writing of the report. The corresponding author had full access to all the data in the study and had final responsibility for the decision to submit for publication.

## Results

**Figure 1:**
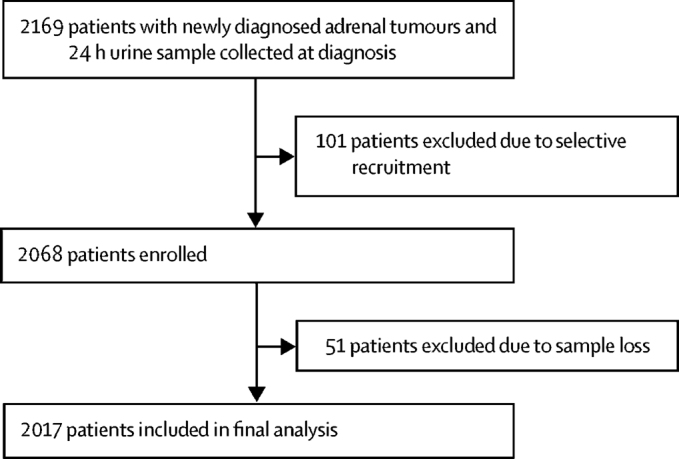
Study profile

Between Jan 17, 2011, and July 15, 2016, 2169 eligible participants were recruited from the 21 participating centres and provided 24 h urine samples (figure 1, appendix p 14). However, review of the data showed that only 14 of the 21 centres had a high median annual recruitment rate (33 participants per year) and a median centre-specific proportion of ACC of 3·9%. These 14 centres recruited a total of 2068 participants. By contrast, the seven remaining centres jointly recruited only 101 participants (median annual recruitment rate 6·7), but the median proportion of ACC cases was 35% (appendix p 21). Investigators at these seven sites confirmed that they had recruited selectively, favouring large and suspicious masses, compared with non-selective consecutive recruitment at the other 14 centres. Therefore, we excluded the 101 participants from these seven centres. An additional 51 participants were excluded from the analysis because of sample loss during storage, transport, or processing; therefore, the final analysis cohort consisted of 2017 participants (figure 1). No participants were lost to follow-up.

The median age of included participants was 59 years, 62% were women, and 84% of the adrenal tumours were discovered incidentally (table 1). Diagnosis by the reference standards was ACC in 98 (4·9%), other malignant tumours in 65 (3·2%), ACA in 1767 (87·6%), and other benign tumours in 87 participants (4·3%; table 1, appendix pp 11, 22). Histopathology results were used to provide the diagnosis for 91 (92·9%) of the ACCs, 65 (100·0%) of other malignant tumours, 370 (20·9%) of ACAs, and 59 (67·8%) of other benign tumours. Clinical and radiological follow-up assessments were used to provide the diagnosis for all remaining tumours (appendix p 23).

**Table 1:** Clinical characteristics and radiological findings of participants included in the study cohort

		**ACC (n=98)**	**Other malignant tumours (n=65)**	**ACA (n=1767)**	**Other benign tumours (n=87)**	**Total (n=2017)**
Sex						
	Men	37 (37·8%)	38 (58·5%)	663 (37·5%)	37 (42·5%)	775 (38·4%)
	Women	61 (62·2%)	27 (41·5%)	1104 (62·5%)	50 (57·5%)	1242 (61·6%)
Age at diagnosis (years)		50 (42–61)	64 (53–70)	59 (50–67)	52·5 (44–62)	59 (49–67)
Incidentally discovered						
	Yes	43 (43·9%)	56 (86·2%)	1513 (85·6%)	74 (85·1%)	1686 (83·6%)
	No*	55 (56·1%)	9 (13·8%)	254 (14·4%)	13 (14·9%)	331 (16·4%)
Location of tumour						
	Right adrenal	38 (38·8%)	23 (35·4%)	556 (31·5%)	43 (49·4%)	660 (32·7%)
	Left adrenal	60 (61·2%)	35 (53·8%)	843 (47·7%)	35 (40·2%)	973 (48·2%)
	Both adrenals	0	7 (10·8%)	368 (20·8%)	9 (10·3%)	384 (19·0%)
Maximum tumour diameter (cm)		9·6 (6·5–13·5)	6·5 (3·7–10·0)	2·5 (1·7–3·5)	4·7 (2·7–7·0)	2·7 (1·8–3·9)
	<2	0	6 (9·2%)	559 (31·6%)	11 (12·6%)	576 (28·6%)
	2 to <4	2 (2·0%)	13 (20·0%)	912 (51·6%)	26 (29·9%)	953 (47·2%)
	≥4	96 (98·0%)	46 (70·8%)	296 (16·8%)	50 (57·5%)	488 (24·2%)
Unenhanced CT tumour attenuation						
	<10 HU†	0	0	905 (51·2%)	23 (26·4%)	928 (46·0%)
	10–20 HU†	1 (1·0%)	1 (1·5%)	223 (12·6%)	9 (10·3%)	234 (11·6%)
	>20 HU‡	32 (32·7%)	38 (58·5%)	200 (11·3%)	30 (34·5%)	300 (14·9%)
	Heterogeneous§	65 (66·3%)	21 (32·3%)	0	1 (1·1%)	87 (4·3%)
	Not done	0	5 (7·7%)	439 (24·8%)	24 (27·6%)	468 (23·2%)
MRI						
	Chemical shift present†	0	1 (1·5%)	247 (14·0%)	5 (5·7%)	253 (12·5%)
	Chemical shift absent‡	1 (1·0%)	8 (12·3%)	65 (3·7%)	15 (17·2%)	89 (4·4%)
	Not done	97 (99·0%)	56 (86·2%)	1455 (82·3%)	67 (77·0%)	1675 (83·0%)
^18^F-FDG PET						
	Low uptake†	0	0	123 (7·0%)	10 (11·5%)	133 (6·6%)
	High uptake‡	2 (2·0%)	17 (26·2%)	4 (0·2%)	5 (5·7%)	28 (1·4%)
	Not done	96 (98·0%)	48 (73·8%)	1640 (92·8%)	72 (82·8%)	1856 (92·0%)
CT absolute contrast washout						
	≥60%†	0	0	19 (1·1%)	0	19 (0·9%)
	<60%‡	0	0	2 (0·1%)	0	2 (0·1%)
	Not done	98 (100·0%)	65 (100·0%)	1746 (98·8%)	87 (100·0%)	1996 (99·0%)
Maximum diameter on CT after ≥6 months						
	Stable or reduced†	0	5 (7·7%)	611 (34·6%)	26 (29·9%)	642 (31·8%)
	Increased >20%‡	0	8 (12·3%)	6 (0·3%)	8 (9·2%)	22 (1·1%)
	Not done	98 (100·0%)	52 (80·0%)	1150 (65·1%)	53 (60·9%)	1353 (67·1%)
Invasive intervention						
	Adrenalectomy	84 (85·7%)	50 (76·9%)	370 (20·9%)	59 (67·8%)	563 (27·9%)
	Biopsy	7 (7·1%)	11 (16·9%)	0	0	18 (0·9%)
	None	7 (7·1%)	4 (6·2%)	1397 (79·1%)	28 (32·2%)	1436 (71·2%)

Data are n (%) or median (IQR). ACC=adrenocortical carcinoma. ACA=adrenocortical adenoma. HU=Hounsfield units. ^18^F-FDG=^18^F-fluorodeoxyglucose.

* Non-incidental modes of discovery included adrenal masses discovered after imaging done for either clinical signs and symptoms indicative of possible steroid excess or suggestive of a tumour (ie, mass effect with abdominal discomfort or symptoms with weight loss, low grade fever, or both).

† Negative for ACC.

‡ Positive for ACC.

§ HU could not be reliably measured and the tumour was classified as positive.

563 (27·9%) of 2017 participants underwent adrenalectomy, including 370 with ACA and 59 with other benign tumours (appendix p 22). 186 of these ACAs (50·3%) were either non-functioning (n=81) or showed only mild autonomous cortisol secretion (n=105). Thus, 245 (43·5%) of 563 surgically managed participants would not have required adrenalectomy. Even if mild autonomous cortisol secretion was used as an indication for adrenalectomy, which is not current clinical practice, 140 (24·9%) of 563 adrenal masses would still have not required adrenalectomy.

**Figure 2:**
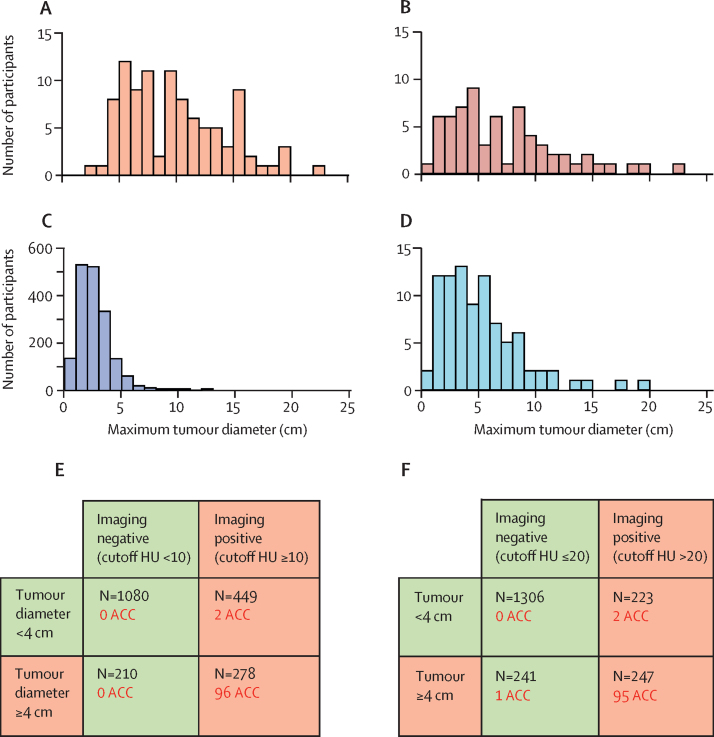
Imaging test results Maximum tumour diameter in patients with ACC (A), other malignant tumours (B), ACA (C), and other benign tumours (D) and distributions of patients with ACC according to positive or negative results for tumour diameter and imaging characteristics with unenhanced CT tumour attenuation cutoff of 10 HU (E) or 20 HU (F). ACA=adrenocortical adenoma. ACC=adrenocortical carcinoma. HU=Hounsfield units.

2737 imaging tests were done in the 2017 participants (appendix p 18). A tumour diameter of at least 4 cm was seen in most participants with ACC and other malignant tumours, around two-thirds of those with other benign tumours and nearly 17% of those with ACA (table 1, figure 2, appendix p 24). The highest-ranking imaging characteristics results were obtained by unenhanced CT in 1549 (76·8%), MRI chemical shift analysis in 227 (11·3%), ^18^F-FDG PET in 43 (2·1%), follow-up CT in 155, and CT contrast washout assessment in six participants (appendix p 18). All 98 participants with ACC had homogeneous tumours with unenhanced CT tumour attenuation of at least 10 HU or heterogeneous tumours precluding HU measurement (table 1). However, among 1328 participants with ACA, unenhanced CT attenuation was greater than the cutoff of 10 HU (ie, false positive) in 423 participants (31·9%; table 1). With attenuation greater than 20 HU, 97 (99%) participants with ACC remained true positive and false-positive results in ACAs decreased to 200 (15·1%). This change in cutoff from 10 HU to 20 HU therefore improved the specificity for ACC from 64·0% (95% CI 61·4–66.4) to 80·0% (77·9–82·0; appendix p 24). Changing the cutoff from 10 to 20 HU improved specificity across the whole study cohort (figure 2, appendix p 24). For the other imaging modalities, false-positive results in ACAs were seen in 65 (20·8%) of 312 participants with MRI, four (3·1%) of 127 with ^18^F-FDG PET, and six (1·0%) of 617 with follow-up CT. Tumour diameter and imaging characteristics yielded similar PPVs and NPVs for ACC (table 2, appendix p 24).

**Table 2:** Performance of tests and combination test strategies

		**ACC (n=98)**	**Other malignant tumours (n=65)**	**All non-ACC tumours (n=1919)**	**ACA (n=1767)**	**Other benign tumours (n=87)**	**Total (n=2017)**	**Percentage of ACC cases (95% CI)**	**Percentage of non-ACC cases (95% CI)**	**Likelihood ratio (95% CI)**	**Post-test probability of ACC (per 100 participants with results)**
**Single-test strategies**											
Tumour diameter											
	≥4 cm	96	46	392	296	50	488	98·0% (92·8–99·8)*	20·4% (18·6–22·3)	4·8 (4·4–5·3)	19·7 (16·2–23·5)
	<4 cm	2	19	1527	1471	37	1529	2·0% (0·2–7·2)	79·6% (77·7–81·4)†	0·03 (0·01–0·10)	0·1 (0·0–0·5)
Imaging characteristics‡											
	Positive	97	63	396	289	44	493	99·0% (94·4–100·0)*	20·6% (18·8–22·5)	4·8 (4·4–5·3)	19·7 (16·3–23·5)
	Negative	1	2	1523	1478	43	1524	1·0% (0·0–5·6)	79·4% (77·5–81·2)†	0·01 (0·00–0·09)	0·1 (0·0–0·4)
Urine steroid metabolomics											
	High risk of ACC	83	7	157	143	7	240	84·7% (76·0–91·2)	8·2% (7·0–9·5)	10·4 (8·7–12·3)	34·6 (28·6–41·0)
	Moderate risk of ACC	13	28	655	578	49	668	13·3% (7·3–21·6)	34·1% (32·0–36·3)	0·39 (0·23–0·65)	1·9 (1·0–3·3)
	Low risk of ACC	2	30	1107	1046	31	1109	2·0% (0·2–7·2)	57·7% (55·4–59·9)	0·04 (0·01–0·14)	0·2 (0·0–0·6)
**Combined-test strategies**											
Tumour diameter and imaging characteristics‡											
	≥4 cm and positive	95	45	152	83	24	247	96·9% (91·3–99·4)*	7·9% (6·8–9·2)	12·2 (10·5–14·3)	38·5 (32·4–44·8)
	<4 cm, negative, or both	3	20	1767	1684	63	1770	3·1% (0·6–8·7)	92·1% (90·8–93·2)†	0·03 (0·01–0·10)	0·2 (0·0–0·5)
Tumour diameter and urine steroid metabolomics											
	≥4 cm and high risk of ACC	82	7	46	33	6	128	83·7% (74·8–90·4)	2·4% (1·8–3·2)	34·9 (25·9–47·1)	64·1 (55·1–72·3)
	≥4 cm and moderate risk of ACC	12	20	130	85	25	142	12·2% (6·5–20·4)	6·8% (5·7–8·0)	1·8 (1·0–3·2)	8·5 (4·4–14·3)
	<4 cm, low risk of ACC, or both	4	38	1743	1649	56	1747	4·1% (1·1–10·1)	90·8% (89·4–92·1)	0·04 (0·02–0·12)	0·2 (0·0–0·6)
Imaging characteristics‡ and urine steroid metabolomics											
	Positive and high risk of ACC	82	6	43	35	2	125	83·7% (74·8–90·4)	2·2% (1·6–3·0)	37·3 (27·4–50·8)	65·6 (56·6–73·9)
	Positive and moderate risk of ACC	13	28	155	97	30	168	13·3% (7·3–21·6)	8·0% (6·9–9·4)	1·69 (1·00–2·87)	7·7 (4·2–12·9)
	Negative, low risk of ACC, or both	3	31	1721	1635	55	1724	3·1% (0·6–8·7)	89·7% (88·2–91·0)	0·03 (0·01–0·10)	0·2 (0·0–0·5)
Tumour diameter, imaging characteristics‡, and urine steroid metabolomics											
	≥4 cm, positive, and high risk of ACC	81	6	25	17	2	106	82·7% (73·7–89·6)	1·3% (0·8–1·9)	63·4 (42·5–94·6)	76·4 (67·2–84·1)
	≥4 cm, positive, and moderate risk of ACC	12	20	58	23	15	70	12·2% (6·5–20·4)	3·0% (2·3–3·9)	4·1 (2·3–7·3)	17·1 (9·2–28·0)
	<4 cm, negative, low risk of ACC, or a combination	5	39	1836	1727	70	1841	5·1% (1·7–11·5)	95·7% (94·7–96·5)	0·05 (0·02–0·13)	0·3 (0·0–0·6)

Data in columns one to six are numbers of participants. ACC=adrenocortical carcinoma. ACA=adrenocortical adenoma.

* Sensitivity.

† Specificity.

‡ Positive was classified as unenhanced CT attenuation >20 Houndsfield units in homogeneous tumours or heterogeneous tumours precluding measurement of attenuation.

The accuracy of urine steroid metabolomics was high (AUROC 94·6%, 95% CI 92·2–96·9; appendix p 29). Urine steroid metabolomics profiles indicating high risk of ACC were seen in 83 (84·7%) of 98 participants with ACC and 157 (8·2%) of 1919 with non-ACC masses (table 2, appendix p 29). High risk of ACC indicated by urine steroid metabolomics substantially improved PPV compared with imaging tests, while a low-risk score had similar ability to imaging tests to rule out ACC (table 2). A urine steroid metabolomics profile indicating moderate risk of ACC (n=668 [33·1%]) was associated with a PPV of 1·9% (table 2).

Cross-tabulation of urine steroid metabolomics results against results obtained with the three most frequently used imaging modalities—unenhanced CT, MRI chemical shift, and ^18^F-FDG PET—showed that high risk of ACC based on urine steroid metabolomics was consistently associated with lower false-positive rates (appendix pp 26–28). Imaging performance was generally better in non-ACC tumours smaller than 4 cm than in those 4 cm or larger. Unenhanced CT had a higher true-positive rate for ACC than a urine steroid metabolomics result indicating high risk of ACC (appendix p 26); MRI or ^18^F-FDG PET were used to assess very few ACCs (appendix pp 27–28), precluding comparisons with these methods.

**Figure 3:**
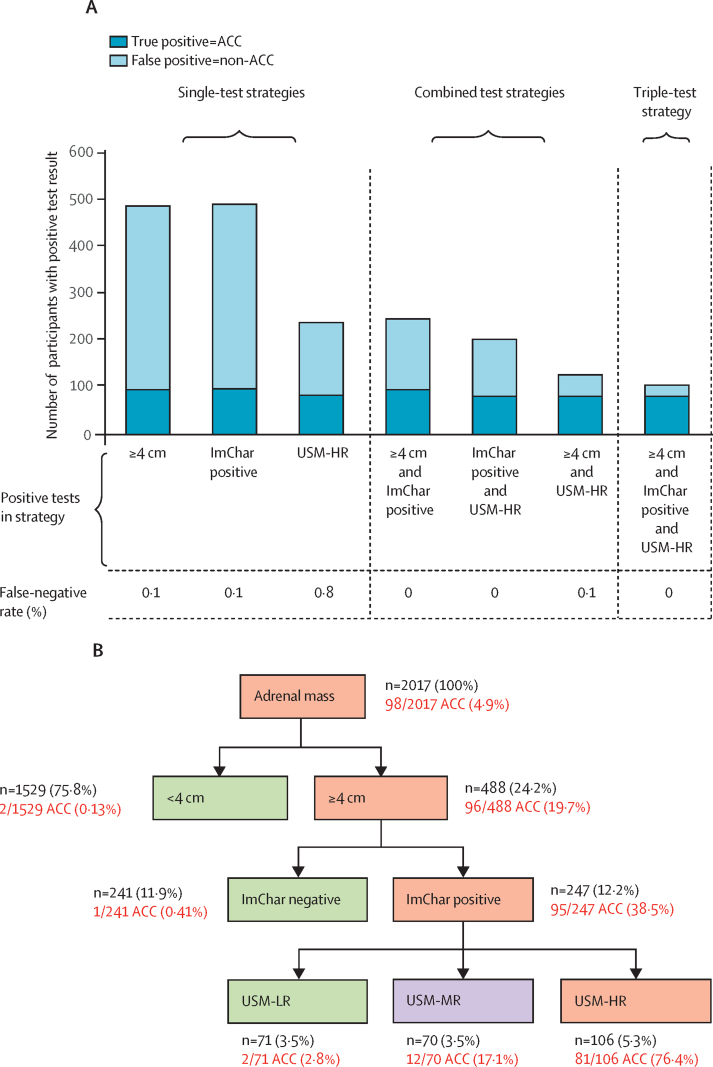
Diagnostic accuracy of single-test and multiple-test strategies for detecting ACC (A) Diagnostic accuracy of the three index tests (tumour diameter, imaging characteristics [unenhanced CI attenuation >20 HU], and urine steroid metabolomics) as single tests, in double combinations, and as a triple-test strategy. (B) Flowchart illustrating the distribution of ACC cases when applying the triple-test strategy in the order: tumour diameter, imaging characteristics (unenhanced CT attenuation >20 HU), and urine steroid metabolomics. ACC=adrenocortical carcinoma. HU=Hounsfield units. ImChar=imaging characteristics. USM-HR=urine steroid metabolomics profile indicating high risk of ACC. USM-LR=urine steroid metabolomics profile indicating low risk of ACC. USM-MR=urine steroid metabolomics profile indicating moderate risk of ACC.

247 (12·2%) of 2017 participants had positive results for tumour diameter and imaging characteristics. Among these, 95 of the 98 ACCs were true positives, leaving 152 false-positive results (figure 3). Participants with a urine steroid metabolomics result indicating a high risk of ACC and a positive result for either tumour diameter or imaging characteristics increased the PPV for ACC compared with having combined positive tumour diameter and imaging characteristic results (table 2, figure 3, appendix p 29). When urine steroid metabolomics was combined with tumour diameter, only four participants with ACC were dismissed, and when combined with imaging characteristics only three were dismissed (table 2).

In the triple testing strategy, a result of tumour diameter larger than 4 cm, positive imaging characteristics (attenuation >20 HU), and a urine steroid metabolomics result indicating high risk of ACC, yielded a group of 106 participants, including 81 of the 98 participants with ACC (figure 3), giving a PPV for ACC of 76·4% (table 2). The triple testing strategy classified 70 participants as having moderate risk of ACC, including 12 of the 98 participants with ACC (PPV 17·1%, table 2). The ability of the triple testing strategy to rule out ACC was high (table 2).

In a sensitivity analysis, when participants with mixed or aberrant steroid excess (n=42), clinical presentation of large adrenal mass with extra-adrenal metastases (n=13), and bilateral macronodular adrenal hyperplasia and isolated cortisol excess (n=22) were excluded, the high accuracy of urine steroid metabolomics compared with imaging was confirmed, as was the improvement of accuracy when urine steroid metabolomics was used in combination with imaging modalities (appendix pp 16, 30–32).

Among the 65 non-ACC malignant tumours, 46 (70·8%) had a tumour diameter greater than 4 cm, 63 (97·0%) had positive imaging characteristics, and seven (10·8%) had a urine steroid metabolomics profile indicating high risk of ACC. These tumours could not be reliably differentiated from ACA and other benign tumours with any of the combined testing strategies (appendix pp 17, 33–35)

## Discussion

Our results have validated the diagnostic utility of urine steroid metabolomics in detecting ACC in participants with newly diagnosed adrenal masses. Diagnostic accuracy of urine steroid metabolomics was high compared with maximum tumour diameter and imaging characteristics, and the best performance was seen when these three methods were combined. We also showed that using a cutoff of 20 HU for unenhanced CT tumour attenuation increases the accuracy of imaging characteristic assessment for exclusion of ACC compared with the currently recommended cutoff of 10 HU, which has immediate implications for clinical practice.

1686 (83·6%) of the adrenal tumours in our study cohort had been discovered incidentally, yielding to our knowledge the largest prospective cohort of participants with adrenal incidentaloma. We achieved this study size via a comprehensive, multicentre, non-selective approach, completing recruitment in 5·5 years. This cohort size compares favourably with the largest retrospective adrenal incidentaloma cohort, in which 1096 patients were identified over a 15-year period (1980–95).^10^ The distribution of underlying pathologies in our cohort was similar to those in retrospective studies,^10^, ^11^ showing the representativeness of our cohort for clinical practice.

96 of 98 ACCs in our study were larger than 4 cm. This cutoff was previously suggested to be sensitive (93%) for differentiating ACC from ACA, but to have poor specificity.^10^, ^11^, ^15^, ^17^, ^22^ Given the non-selective nature of recruitment in EURINE-ACT and the small number of ACCs less than 4 cm in diameter, growth velocity of these tumours seems to be very rapid, making early-stage detection unlikely. 1549 (76·8%) of participants in the study underwent unenhanced CT. Only one ACC had attenuation below 20 HU, giving this cutoff considerably improved specificity compared with the 10 HU cutoff recommended in the European guidelines.^11^, ^15^, ^16^, ^17^ Applying cutoffs of 4 cm for maximum tumour diameter and 20 HU for unenhanced CT tumour attenuation would, therefore, help to avoid unnecessary imaging procedures and adrenalectomies.

PPV was greater with urine steroid metabolomics than with tumour diameter and imaging characteristics. Combination of all three approaches gave the best overall detection of ACC, even when ACC tumours potentially identifiable by other parameters, such as mixed or aberrant steroid patterns or clinical presentations, were excluded. Based on our triple test strategy in the whole study cohort (n=2017), following the initial scan that provided tumour size measurements, only 488 participants (24·2%) would have required further imaging. Additionally, participants with a urine steroid metabolomics result indicating high risk of ACC could have undergone surgery for ACC earlier and fewer unnecessary surgeries for benign tumours would have been done. For participants with urine steroid metabolomics results indicating moderate risk of ACC in the triple strategy, the PPV was 17%. Rather than blanket surgical removal, these participants could be managed individually, such as with detailed re-review of scans by a multidisciplinary team and consideration of biopsy. Although histopathology of an adrenal biopsy cannot differentiate between ACC and ACA, it can be informative in patients with other benign and malignant adrenocortical tumours, including metastases of extra-adrenal cancers.

Strengths of our study include its prospective, consecutive, and non-selective recruitment, and the relatively short time needed for recruitment (avoiding biases due to change in diagnostic technologies and standards). Our previous proof-of-principle study of urine steroid metabolomics for ACC detection involved urinary steroid profiling by gas chromatography-mass spectrometry, which is a low-throughput method requiring highly specialised expertise.^18^ In this study, we used the high-throughput LC–MS/MS approach, which is much more widely available. Although a similar multisteroid profiling method has been described elsewhere,^23^ our method includes a machine-learning algorithm that was trained on steroid data measured by LC–MS/MS in a retrospective cohort, precluding error due to non-standardised assessment of steroid profiling results. Other strengths of our study include the exclusion of participants undergoing imaging for cancer monitoring and the non-communication of urine steroid metabolomics results to clinical centres so as not to influence the usual diagnostic process.

Weaknesses of this study include its observational nature; notably, recruitment centres could use their preferred imaging modalities and, therefore, the numbers assessed by MRI or ^18^F-FDG PET were too low for comprehensive assessment. Participating centres were specialist adrenal tumour centres, but in non-specialised secondary care settings the proportions of large and malignant tumours might be lower. Histopathology was done only for participants who had surgery or biopsy, meaning that our reference standard was heterogeneous. However, it was of higher quality than most earlier diagnostic studies^15^, ^24^ and is representative of clinical practice. We did not do a centralised pathology review, but as the study sites were high-volume specialist centres with established adrenal pathology expertise, all pathologists applied relevant multifactorial scoring systems for assessment of malignant potential in adrenal cortical neoplasms.^25^, ^26^

In conclusion, our findings suggest that use of urine steroid metabolomics in diagnostic pathways could improve detection of ACC. We recommend a combined testing strategy of assessment with unenhanced CT, with a tumour attenuation cutoff of 20 HU, and urine steroid metabolomics. We anticipate that these changes would substantially lessen the burden on and morbidity in patients with benign adrenal tumours and suspicious imaging findings, and lead to reductions in health-care costs due to decreased numbers of imaging procedures, time to surgery in ACC, and numbers of unnecessary surgeries.

## Data sharing

We will consider sharing de-identified, individual participant-level data that underlie the results reported in this Article on receipt of a request detailing the study hypothesis and statistical analysis plan. All requests should be sent to the corresponding author. The corresponding author and lead investigators of this study will discuss all requests and make decisions about whether data sharing is appropriate based on the scientific rigour of the proposal. All applicants will be asked to sign a data access agreement.
