# Supplementary material for: Urine steroid metabolomics for the differential diagnosis of adrenal incidentalomas in the EURINE-ACT study: a prospective test validation study
Source: Lancet Diabetes Endocrinol. 2020 Sep;8(9):773–81. doi: 10.1016/S2213-8587(20)30218-7 (PMC7447976; doi:10.1016/S2213-8587(20)30218-7)
Supplement: Supplementary appendix [file mmc1.pdf]

THE LANCET
Diabetes & Endocrinology

Supplementary appendix

This appendix formed part of the original submission and has been peer reviewed. We post it as supplied by the authors.

Supplement to: Bancos I, Taylor A E, Chortis V, et al. Urine steroid metabolomics for the differential diagnosis of adrenal incidentalomas in the EURINE-ACT study: a prospective test validation study. *Lancet Diabetes Endocrinol* 2020; published online July 23. https://doi.org/10.1016/S2213-8587(20)30218-7.

Supplementary Appendix

"Urine steroid metabolomics in the differential diagnosis of adrenal incidentalomas: A prospective test validation study"

List of ENSAT EURINE-ACT Investigators

(listed in alphabetical order by country and institution)

Australia

- School of Computing and Information, University of Melbourne, Melbourne, Australia (Stephan Glöckner, Richard O. Sinnott, Anthony Stell)

Brazil

- Adrenal Unit, Divison of Endocrinology and Metabolism, Hospital das Clinicas, University of São Paulo Medical School, Institute of Cancer of São Paulo, São Paulo Brazil (Maria Candida B. V. Fragoso)

Croatia

- Department of Endocrinology, University Hospital Centre Zagreb, Zagreb, Croatia (Darko Kastelan, Ivana Dora Pupovac, Bojana Simunov)

France

- Department of Endocrinology, Hôpital Haut Lévêque, CHU de Bordeaux, Pessac, France (Sarah Cazenave, Magalie Haissaguerre, Antoine Tabarin)
- National Expert Centre for Rare Adrenal Cancers, Covhin Hospital, Institut Cochin, Institut National de la Santé et de la Recherche Medicale Unite 1016, René Descartes University, Paris (Jérôme Bertherat, Rossella Libé)

Germany

- Endocrinology in Charlottenburg, Berlin, Germany (Tina Kienitz, Marcus Quinkler)
- Institute of Clinical Chemistry and Laboratory Medicine, University Hospital Carl Gustav Carus, Technical University, Dresden, Germany (Katharina Langton, Graeme Eisenhofer)
- Medizinische Klinik and Poliklinik IV, Ludwig-Maximilians-Universität München, Munich, Germany (Felix Beuschlein, Christina Brugger, Martin Reincke, Anna Riester, Ariadni Spyroglou)
- Division of Endocrinology and Diabetes, Department of Internal Medicine I, University Hospital, University of Würzburg, German and Comprehensive Cancer Centre Mainfranken, University of Würzburg, Würzburg, Germany (Stephanie Burger-Stritt, Timo Deutschbein, Martin Fassnacht, Stefanie Hahner, Matthias Kroiss, Cristina L. Ronchi)

Greece

- Department of Endocrinology, Diabetes and Metabolism, Evangelismos Hospital, Athens, Greece (Sotiria Palimeri, Stylianos Tsagarakis, Ioanna Tsirou, Dimitra Vassiliadi)

Italy

- Department of Clinical and Biological Sciences, San Luigi Hospital, University of Turin, Turin, Italy (Vittoria Basile, Elisa Ingargiola, Giuseppe Reimondo, Massimo Terzolo)
- Department of Experimental and Clinical Biomedical Sciences, University of Florence, Florence, Italy (Letizia Canu, Massimo Mannelli)

The Netherlands

- Department of Internal Medicine, Maxima Medisch Centrum, Eindhoven, The Netherlands (Hester Ettaieb, Harm R. Haak, Thomas M. Kerkhofs)
- Department of Health Services Research, and CAPHRI School for Public Health and Primary Care, Maastricht University, The Netherlands (Harm R. Haak)
- Bernoulli Institute for Mathematics, Computer Science and Artificial Intelligence, University of Groningen, Groningen, The Netherlands (Michael Biehl)
- Department of Internal Medicine, Division of Endocrinology, Erasmus Medical Centre, University Medical Centre Rotterdam, Rotterdam, The Netherlands (Richard A. Feelders, Johannes Hofland, Leo J. Hofland)

Norway

- Department of Clinical Science, University of Bergen, and Department of Medicine, Haukeland University Hospital, Bergen, Norway (Marianne A. Grytaas, Eystein S. Husebye, Grethe A. Ueland)

Poland

- Department of Internal Medicine and Endocrinology, Medical University of Warsaw, Warsaw, Poland (Urszula Ambroziak, Tomasz Bednarczuk, Agnieszka Kondracka, Magdalena Macech, Malgorzata Zawierucha)

Portugal

- Department of Endocrinology, University Hospital of Coimbra, Coimbra, Portugal (Isabel Paiva)

Republic of Ireland

- School of Medicine, National University of Ireland Galway (NUIG), Galway, Republic of Ireland (M. Conall Dennedy, Ahmed Sajwani)
- Department of Endocrinology, Beaumont Hospital, Dublin, and the Royal College of Surgeons in Ireland, Dublin, Republic of Ireland (Mark Sherlock)
- Department of Endocrinology, St. Vincent's University Hospital, Dublin, and School of Medicine, University College Dublin, Dublin, Republic of Ireland (Rachel K. Crowley)

Serbia

- Department for Obesity, Reproductive and Metabolic Disorders, Clinic for Endocrinology, Diabetes and Metabolic Diseases, Clinical Centre of Serbia, Faculty of Medicine, University of Belgrade, Belgrade, Serbia (Miomira Ivovic, Ljiljana Marina)

United Kingdom

- Institute of Applied Health Research, University of Birmingham, Birmingham, UK (Jonathan J. Deeks, Alice J. Sitch)
- Institute of Metabolism and Systems Research, University of Birmingham, and Centre for Endocrinology, Diabetes and Metabolism, Birmingham Health Partners, Birmingham, UK (Wiebke Arlt, Irina Bancos, Vasileios Chortis, Lorna C. Gilligan, Beverly A. Hughes, Katharina Lang, Hannah E. Ivison, Carl Jenkinson, Konstantinos Manolopoulos, Donna M. O'Neil, Michael W. O'Reilly, Thomas G. Papathomas, Alessandro Prete, Cristina L. Ronchi, Cedric H.L. Shackleton, Angela E. Taylor)
- Department of Endocrinology, Queen Elizabeth Hospital, University Hospitals Birmingham NHS Foundation Trust, Birmingham, UK (Wiebke Arlt, Miriam Asia, Vasileios Chortis, Katharina Lang, Konstantinos N. Manolopoulos, Michael W. O'Reilly, Alessandro Prete, Cristina L. Ronchi)
- Department of Hepato-Pancreato-Biliary and Liver Transplant Surgery, Queen Elizabeth Hospital, University Hospitals Birmingham NHS Foundation Trust, Birmingham, UK (Robert P. Sutcliffe)
- Department of Radiology, Queen Elizabeth Hospital, University Hospitals Birmingham NHS Foundation Trust, Birmingham, UK (Peter Guest)
- Department of Pathology, Queen Elizabeth Hospital, University Hospitals Birmingham NHS Foundation Trust, Birmingham, UK (Kassiani Skordilis)

United States of America

- Division of Endocrinology, Metabolism and Nutrition, Mayo Clinic, Rochester, MN, USA (Irina Bancos, Cristian Bancos, Alice Chang, Caroline J. Davidge-Pitts, Danae A. Delivanis, Dana Erickson, Neena Natt, Todd B. Nippoldt, Melinda Thomas, William F. Young Jr.)
- UCSF Benioff Children's Hospital Oakland Research Institute, Oakland, California, CA, USA (Cedric H.L. Shackleton)

Supplementary Methods

Sample size calculation

The study had a recruitment target of 2000 participants at an expected ACC rate of 5%, determined by sample size calculations based on the results of the proof-of-principle study[1]. Based on a conservative estimate that 5% of tumours would be ACC, observing 100 ACC cases would allow a sensitivity of 95% to be estimated with a 95% confidence interval of width less than 10%. This sample size would have over 99% power to detect a difference of 3% (87% vs 90% assuming positive correlation of errors) in specificity at the 5% significance level, allowing for 10% loss to follow-up, thus would provide ample precision to be able to estimate benefits through reduced false positives. The prevalence of ACC in the final data was 4.9% (98/2017). Final recruitment was 2169 with 2017 used in the final analysis, due to exclusion of selectively recruited patients and samples lost during processing, storage or transport.

Patient recruitment

The European Network for the Study of Adrenal Tumours (ENSAT) database (https://registry.ensat.org) was established in 2008 and serves as a complete virtual research environment for adrenal tumour research, with password protected access to the database by all registered full ENSAT members who have approval of their local ethics committee. Access to the ENSAT database occurs through a unified, security-driven portal that allows targeted upload of pseudonymised patient data. In the EURINE-ACT study, all clinical data were prospectively collected and recorded in the ENSAT registry. Variables included demographic data, mode of adrenal tumour discovery and clinical presentation, tumour diameter and imaging characteristics of the adrenal mass, results of endocrine testing, clinical and radiological follow up data, data on surgery and histopathology, and availability of biomaterial. For bilateral adrenal masses, we selected the larger tumour diameter and the more unfavorable imaging characteristic for analysis. Histopathology and radiological assessment were carried out and recorded locally; all participating referral centres had local access to specialist radiologists and histopathologists highly experienced in the diagnosis and differential diagnosis of adrenal tumours. A diagnosis of ACC was made based on multifactorial scoring systems set out for the diagnosis of adrenal cortical carcinoma in the WHO Classification of Tumours of Endocrine Organs[2]

including a Weiss Score of 3 or above for conventional adrenocortical carcinomas, in accordance with the Histopathology Reporting Guide for Carcinoma of the Adrenal Cortex by the International Collaboration on Cancer Reporting[3].

Prior to enrolment in EURINE-ACT, all participants underwent biochemical exclusion for the presence of pheochromocytoma[4]. All participants underwent standardised endocrine assessment for exclusion of clinically overt Cushing's syndrome and primary aldosteronism, which were diagnosed according to standard guidelines[5,6]. We recorded the presence of mild autonomous cortisol secretion secretion in patients with (1) a lack of clinical features indicative of overt Cushing's syndrome (e.g. proximal myopathy, dorsocervical and supraclavicular fat pads, broad striae), and (2) failure to suppress morning serum cortisol to less than 50 nmol/L (1.8 µg/dL) after administration of 1mg dexamethasone orally at 11 pm the preceding night (1mg-dexamethasone suppression test), as defined by recent guidelines[7].

Each patient provided a 24-hour urine sample and the volume of the 24-h collection was recorded. The samples were aliquoted in the recruitment centre on the day of collection and stored locally at -20°C before transport on dry ice to the University of Birmingham, UK, for mass spectrometry analysis in the Steroid Metabolome Analysis Core of the Institute of Metabolism and Systems Research. Upon receipt, samples were catalogued and compared to the sample list sent by the local recruitment centre and transferred to -20°C storage until analysis. 24-h urines were accepted as accurately collected if their total collection volume was >1000mL and/or if 24-h urinary creatinine excretion was within the reference range.

Urinary steroid metabolite profiling by liquid chromatography-tandem mass spectrometry

From each 24-h urine collection, we aliquoted 400µL of urine and added 40µL of internal standard solution (10µg/mL), containing deuterated steroid standards (DHEA-d6, Cortisol-d4 [Sigma Aldrich, Gillingham, UK]; Etio-d5, THE-d5, THS-d5 [Isosciences, Ambler, USA]). To negate dilution effects, if the the 24-h urine collection volume exceeded 2500mL, we increased the sample volume to 800µL of urine. Samples were then hydrolysed to release the steroids from their sulfate and glucuronide conjugates, after addition of 440µL deconjugation mixture, containing 0.2M acetate buffer (prepared at pH 4.8-5.0), 3.3 mg/mL ascorbate, and 67 U/mL of a sulfatase/glucuronidase enzyme mix derived from

helix pomatia (Sigma Aldrich, Gillingham, UK), followed by heating at 60°C for 3 hours. Thereafter, the solution was allowed to cool, followed by solid phase extraction using Sep Pak C18 cartridges (96-well plate 100mg sorbent per cartridge [Biotage, Hengoed, UK]). The cartridges were washed with 1mL LC-MS grade methanol (Greyhound Chromatography, Birkenhead, UK) and 1mL LC-MS grade water (Fisher Scientific, Loughborough, UK). Next, the urine sample was passed over the cartridge and a further wash was performed with 1mL LC-MS grade water. Following this, steroids were eluted with 1mL of LC-MS grade methanol. This steroid fraction was dried under nitrogen at 55°C and reconstituted in 125µL of 50/50 LC-MS grade methanol/water, vortexed for 5 minutes and centrifuged at 1793 x g prior to mass spectrometry analysis.

A Waters Xevo mass spectrometer with an acquity ultra high performance (uPLC) chromatography system with a HSS T3, 1.8µm, 1.2x50mm column (heated at 60°C) was used to analyse the steroids. A 20µL sample injection volume was used. A mobile phase of LC-MS grade methanol and water, both with 0.1% formic acid was used. Elution of steroids was achieved at a flow rate of 600µL per minute, which started at 45% methanol held for one minute, followed by a linear gradient to 80% methanol at 8.5 minutes. The column was then washed at 98% methanol and re-equilibrated at the starting gradient prior to the next injection.

All 15 steroids (**Suppl. Fig. 2**) were detected in positive ionisation mode. For positive identification and quantification of a steroid, the analyte had to have two matching multiple reaction monitoring (MRM) mass transitions (precursor/product transitions) and an identical retention time relative to an authentic steroid standard. Steroids were quantified compared to a calibration series using standard concentrations of each steroid standard ranging from 10 to 5000ng/mL, with inclusion of a blank, prepared in steroid free synthetic urine matrix (Sigma, Gillingham, UK) and processed as above. Each steroid concentration was calculated relative to an assigned internal standard. Prior to analysis, we had validated the method assessing specificity, sensitivity, accuracy, precision, linearity, limit of detection (LOD), limit of quantification (LOQ), reproducibility, absolute recovery, and matrix effects (**Suppl. Table 3**).

Machine learning and classification algorithm

In order to obtain a classifier system for the discrimination of ACC from ACA, we had analysed an independent, retrospectively collected data set comprising 24-h urine steroid excretion data from 139 patients (99 ACA, 40 ACC) (Taylor AE et al., unpublished, available on request). Steroid excretion in those 139 retrospectively collected patients had been measured by the same LC-MS/MS method employed in this study, yielding values for 15 distinct urinary steroid metabolites (**Suppl. Table 2**), including seven steroids previously described as part of the "malignant steroid fingerprint" identified by machine learning analysis of steroid data obtained by gas chromatography-mass spectrometry[1]. All steroid excretion values were log-transformed and subsequently z-score normalised with respect to the means and standard deviations observed in the data set. The resulting set of 15-dimensional vectors $\mathbf{x}=(x_1,x_2,\dots,x_{15})$, together with the class membership served as input for the machine learning analysis. We employed a variant of Learning Vector Quantization (LVQ)[8] for the computational analysis, Generalised Matrix Relevance LVQ (GMLVQ)[9-11], which represents classes in terms of typical prototypes $\mathbf{w}^{ACA}$ and $\mathbf{w}^{ACC}$. For the comparison of a specific vector $\mathbf{x}$ with a prototype $\mathbf{w}=(w_1,w_2,\dots,w_{15})$, a distance measure of the form $d(\mathbf{x},\mathbf{w})= \Sigma_{i,j}\ (x_i-w_i)\ \Lambda_{ij}\ (x_j-w_j)$ is employed. The prototypes $\mathbf{w}^{ACA}$ and $\mathbf{w}^{ACC}$ as well as the matrix $\mathbf{\Lambda}$ of coefficients Λ_{ij} are determined in a cost function based training process; mathematical details of this approach have been previously described[10-12] and it has been applied to multi-steroid data in our urine steroid metabolomics proof-of-principle study[1]. We employed a publicly available implementation of GMLVQ using default parameters[9]. The training process was repeated for 1000 randomly selected subsets of 90% of the data and $\mathbf{w}^{ACA}$, $\mathbf{w}^{ACC}$ and $\mathbf{\Lambda}$ were obtained as averages over these 1000 runs.

In contrast to other machine learning algorithms, GMLVQ yields an interpretable, white box classification algorithm[10]. Numerical values of the coefficients w_i^{ACA}, w_i^{ACC} and Λ_{ij} as well as the parameters of the z-score transformation are available upon request from the corresponding author.

In this paper, we followed to a large extent the set-up and methodology of our previous analysis of GC-MS steroid data[1], in which we provided proof-of-principle for the urine steroid metabolomics approach and also demonstrated that the GMLVQ approach was superior to other statistical or machine learning approaches, namely logistic regression or linear discriminant analysis (LDA).

Similar to our paper utilizing GMLVQ analysis of GC-MS steroid excretion data[1], machine learning analysis of the 24-h urinary steroid metabolite excretion in the retrospective cohort (99 ACA, 40 ACC) identified the 11-deoxycortisol metabolite THS as the steroid most relevant for the differentiation of ACC from ACA, followed by the pregnenolone and 17-hydroxypregnenolone metabolites 5-PD and 5-PT; however, the diagnostic algorithm used for interpretation of the steroid excretion data in this paper employed the data of all 15 urinary steroid metabolites measured by LC-MS/MS (**Suppl. Table 2**) for diagnostic classification.

A new sample, not contained in the training set, is to be classified according to the following prescription:

- The steroid metabolite excretion data is log-transformed and z-score normalised with the (publicly available) means and standard deviations obtained from the training data. This yields a vector **y** repesenting the sample.
- With the (publicly availble) parameters of the classifier $\mathbf{w}^{\mathbf{ACA}}$, $\mathbf{w}^{\mathbf{ACC}}$ and $\mathbf{\Lambda}$, the quantities $d(\mathbf{y},\mathbf{w}^{\mathbf{ACA}})= \Sigma_{i,j}\ (y_i-w_i^{ACA})\ \Lambda_{ij}\ (x_j-w_j^{ACA})$ and $d(\mathbf{y},\mathbf{w}^{\mathbf{ACC}})= \Sigma_{i,j}\ (y_i-w_i^{ACC})\ \Lambda_{ij}\ (x_j-w_j^{ACC})$ are computed.
- The corresponding score is given as $s(\mathbf{y})=1/(1+\exp([d(\mathbf{y},\mathbf{w}^{ACC})-d(\mathbf{y},\mathbf{w}^{ACA})]/10))$, which satisfies $0< s(\mathbf{y})<1$.

Along these lines, the resulting classifier system was applied to the urine steroid metabolite excretion data from the prospective EURINE-ACT cohort. A particular binary GMLVQ classifier can be specified by considering a threshold value θ and assigning data with $s(\mathbf{y})<= \theta$ to class ACA, while samples with $s(\mathbf{y})> \theta$ are classified as ACC. By variation of the threshold parameter, θ-dependent sensitivities and specificities and, therefore, the full Receiver Operating Characteristics (ROC) can be determined[13] (**Suppl. Fig. 2**).

Machine learning-based classifier system and definition of risk thresholds (ACC vs. Non-ACC)

Next, we defined urine steroid metabolomics (USM) score thresholds in order to assign a sample to one of three classes: high risk (USM-HR), moderate risk (USM-MR), and low risk (USM-LR) of ACC. The corresponding thresholds were selected to ensure that the post-test probability of ACC in the high risk group was at least 65% and at least 10% in the moderate risk group. These cut-offs were obtained through a modified Delphi process involving 21 clinicians from 12 countries; all of them are members of the ENSAT EURINE-ACT Investigator group and have long-standing expertise in the management of patients with adrenal tumours including ACC. The experts were asked to identify the post-test probabilities that in their opinion were required to confidently recommend surgery (high risk group), individualised management with surgery or biopsy (moderate risk group), or no further treatment (low risk group). These cut-offs were then applied to the USM scores obtained from the prospective EURINE-ACT data.

Additional machine learning-based classifier system to differentiate four types of adrenal masses (ACC, ACA, OM, OB)

Our machine learning-based classifier was designed and developed to solve a two-class problem, i.e. differentiate ACC from ACA. However, following completion of the ENSAT EURINE-ACT recruitment, we realised that non-selective recruitment of a very large number of patients actually results in four classes of adrenal masses: ACC, ACA, but also other benign (OB) and other malignant (OM) adrenal masses. Therefore, we trained an additional multi-class GMLVQ system aiming at the discrimination of these four classes (ACA, OB, ACC, OM) represented by one prototype each. This was done by selecting class-balanced random subsets of 65 samples from each class from the prospective data for training and determination of the average performance of the obtained system over 50 such randomised training processes. Analogously, a further classifier was obtained by considering only ACA, OB, and OM samples. Results of this post hoc analysis using both of these classifiers are described in the **Supplementary Results** section and displayed in **Suppl. Fig. 4**.

Diagnostic strategy

Each of the index tests (tumour size, imaging characteristics, USM risk score) was considered individually and as a test strategy in combination with one or both other index tests. Strategies including tumour diameter use the diameter as the first test. Participants with tumour diameter above the threshold of 4cm and/or positive imaging characteristics were classed as 'high risk of ACC' if they had a high urine steroid metabolomics score and as 'moderate risk of ACC' if they had a moderate urine steroid metabolomics score. Participants with a tumour diameter below the threshold and/or negative imaging and/or low urine steroid metabolomics score were considered 'low risk of ACC'. As outlined in the above section on the definition of ACC risk thresholds, the urine steroid metabolomics score cut-offs were applied according to the outcome of a modified Delphi consensus to achieve post-test probability ACC in line with the expectation of the 21 expert clinicians who participated in the Delphi process. Secondary analyses evaluated alternative strategy combinations identifying participants with malignant adrenal masses other than ACC (other malignant, OM).

Statistical analysis

Characteristics of participants were described for each target condition defined by the reference standard, with data for continuous and categorical analysis presented as median [lower and upper quartiles] and n (%) respectively. For each test, results were tabulated against the reference standard diagnosis: ACC and non-ACC, as well as for the subcategories of non-ACC of ACA (adrenocortical adenoma); OB (Other Benign); and OM (Other Malignant). For each index test, we computed the percentage of ACC cases with each test result (giving sensitivity for a positive result for a binary test); the percentage of non-ACC cases with each test result (giving specificity for a negative result for a binary test); and the likelihood ratio for each test result. We also computed the proportion with ACC with each test result to estimate the probability of ACC (the positive predictive value [for positive test result], 1 - negative predictive value [for negative test results]). All results were expressed using 95% confidence intervals, computed using the exact binomial method for proportions and using wald based methods for likelihood ratios.

Supplementary Results

Results of endocrine function assessment

Of the 1767 participants with benign adrenocortical adenomas (ACA), 913 (51.7%) showed no evidence of adrenal hormone excess based on the assessments carried out at the clinical recruitment centres (**Suppl. Table 5**). Primary aldosteronism was diagnosed in 153 (8.7%) ACAs, with N=118 (77.1%) diagnosed non-incidentally, mostly due to treatment-resistant or hypokalemic hypertension. By contrast, the majority of the 77 patients with overt adrenal Cushing's syndrome were diagnosed incidentally (N=46, 59.7%) (**Suppl. Table 5**). Mild autonomous cortisol secretion was diagnosed in 602 participants with ACA, with incidental discovery of the adrenal mass in 95% (**Suppl. Table 5**).

Fourty-five (46%) of the 98 ACC patients presented with clinical signs and symptoms of adrenal hormone excess; however, results of routine biochemistry showed evidence of hormone excess in 76 patients (78%). Isolated glucocorticoid and adrenal androgen excess was documented in 18 and 13 patients, respectively. Combined glucocorticoid and adrenal androgen excess was documented in 34 patients. Eight ACC patients had evidence of aldosterone excess and 13 had 17β-estradiol excess.

Though all patients had undergone exclusion of pheochromocytoma prior to inclusion in EURINE-ACT, histopathology revealed 10 patients with phaeochromocytoma, which were biochemically silent, i.e. not detected by plasma or urinary metanephrine analysis.

Post hoc analysis of diagnostic accuracy in a more stringently selected patient cohort

To create an even more stringently selected patient cohort for an additional post hoc analysis, we analysed the pattern of steroid excess, as assessed by routine serum biochemistry, and the clinical and radiological presentation to identify those patients who were readily identifiable as ACC or ACA based on these parameters (**Suppl. Fig. 3**). We defined patients identifiable as ACC (**Suppl. Table 13**) as those who had either a steroid excess pattern that was aberrant, i.e. not of typical adrenal origin (e.g. estradiol), or mixed steroid excess (any combination of steroid classes other than glucocorticoid and mineralocorticoid co-secretion, which is also regularly observed in benign adrenal tumours [1,12]. In addition, we considered patients presenting with a large adrenal mass and extra-adrenal metastases as likely ACC (**Suppl. Table 13**). Similarly, we excluded 22 ACA patients from the post hoc analysis

cohort, as they had bilateral macronodular adrenal hyperplasia with isolated cortisol excess and, thus, were readily identifiable as benign (**Suppl. Fig. 3** and **Suppl. Table 13**). The results of the post-hoc analysis of this even more stringently selected patient cohort (N=1940, including 43 ACC) again revealed a higher positive predictive value for urine steroid metaoblomics as compared to routinely used imaging tests (**Suppl. Table 14**).

Adrenal masses other than ACC and ACA

In addition to 98 ACC (4.9%) and 1767 ACA (87.6%), the EURINE-ACT cohort comprised 87 participants (4.3%) with a benign adrenal mass other than ACA (other benign, OB) and 65 participants (3.2%) with a malignant adrenal mass other than ACC (other malignant, OM). Bilateral adrenal masses were diagnosed in 368 ACA, 9 OB, and 7 OM tumours; the EURINE-ACT cohort did not comprise any bilateral masses with different underlying pathologies.

Surgical removal of the adrenal mass was performed in 50 of 65 OM tumours (77%) and in 59 of 87 OB tumours (68%) (**Suppl. Table 5**). Discovery of the adrenal mass upon follow-up imaging carried out as part of screening or monitoring of a previously known non-adrenal malignancy was an exclusion criterion for EURINE-ACT participation. However, histopathology (either following adrenalectomy or biopsy) revealed adrenal metastases of non-adrenal primary tumours in 39 of the 65 OM tumours (60%) (**Suppl. Tables 5 and 6**); this was almost equally split between metastases of a subsequently newly diagnosed non-adrenal primary tumour, and the late occurrence of metastases of a previously diagnosed non-adrenal primary tumour for which the patient no longer underwent follow-up monitoring.

The majority of the malignant masses other than ACC had positive imaging characteristics (63 of 65, 97%), most had a tumour diameter greater than 4cm (46 of 65, 71%), and only a minority had a USM-HR score (7 of 65, 11%). OM tumours could not be reliably differentiated from the other two Non-ACC classes, ACA and OB tumours, with any of the combined test strategies (**Suppl. Tables 15-17**).

The EURINE-ACT study was designed to validate a GMLVQ algorithm that had been developed to address a two class problem, the differentiation of malignant ACC from benign ACA. However, the final analysis cohort of 2017 patients also comprised 65 OM and 87 OB tumours, hence two additional classes. Therefore, we used the prospective data to train two additional algorithms, differentiating

between all four tumour classes (ACC, ACA, OM, OB); this showed excellent separation of the ACC group prototype from the prototype of the other three classes, which, however, appeared indistinguishable (**Suppl. Figure 4A**). We then trained a three class algorithm for the differentiation of the three Non-ACC classes (ACA, OM, OB). This algorithm achieved some degree of separation between ACA, OM, and OB prototypes, with 42-50% of the respective group participants correctly identified (**Suppl. Figure 4B**); however, this performance would be deemed insufficient for use as a clinically relevant diagnostic test.

Supplementary Figures

Suppl. Fig. 1: Recruitment of the ENSAT EURINE-ACT Final Analysis Cohort over the 66-month prospective recruitment period (January 2011 to July 2016). The graphs depict cumulative recruitment (Panel A) and annual recruitment (Panel B) as well as number of recruiting centres per year (Panel C) for the final EURINE-ACT analysis cohort (N=2017) recruited by 14 ENSAT centres from 11 countries.

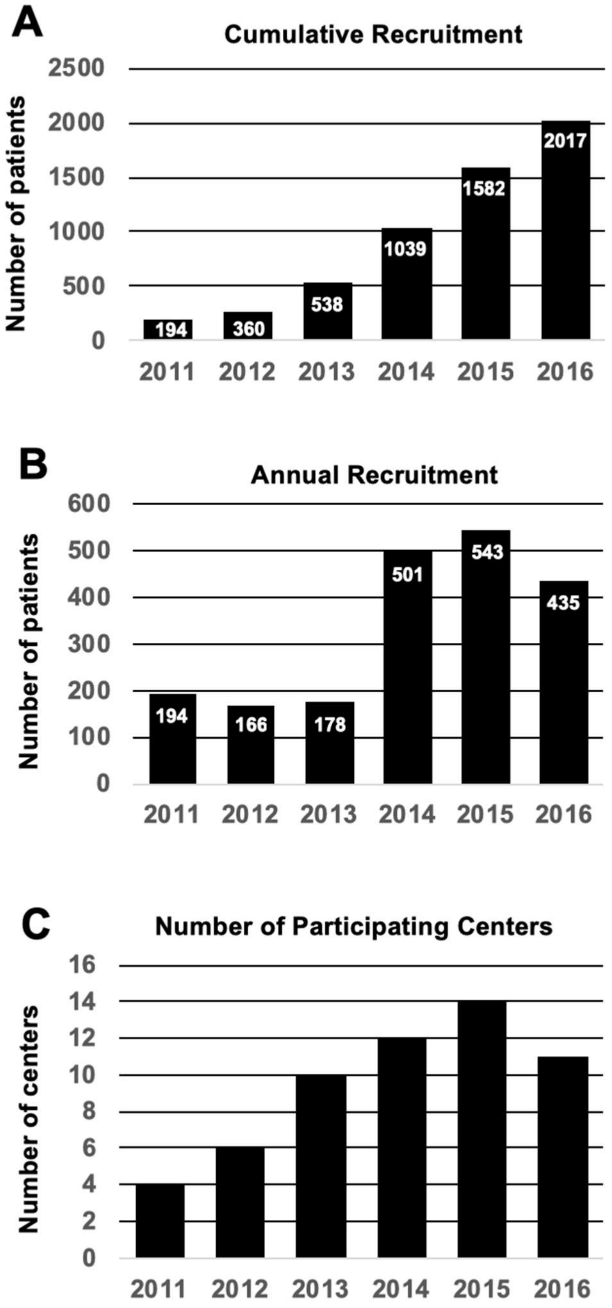

.

Suppl. Fig. 2: Receiver operating characteristic (ROC) curve illustrating the accuracy of urine steroid metabolomics (USM) in detecting ACC. Area Under the ROC curve (AUROC) provided as median and 95% confidence interval, taking into account all 15 urinary steroid metabolites measured by LC-MS/MS.

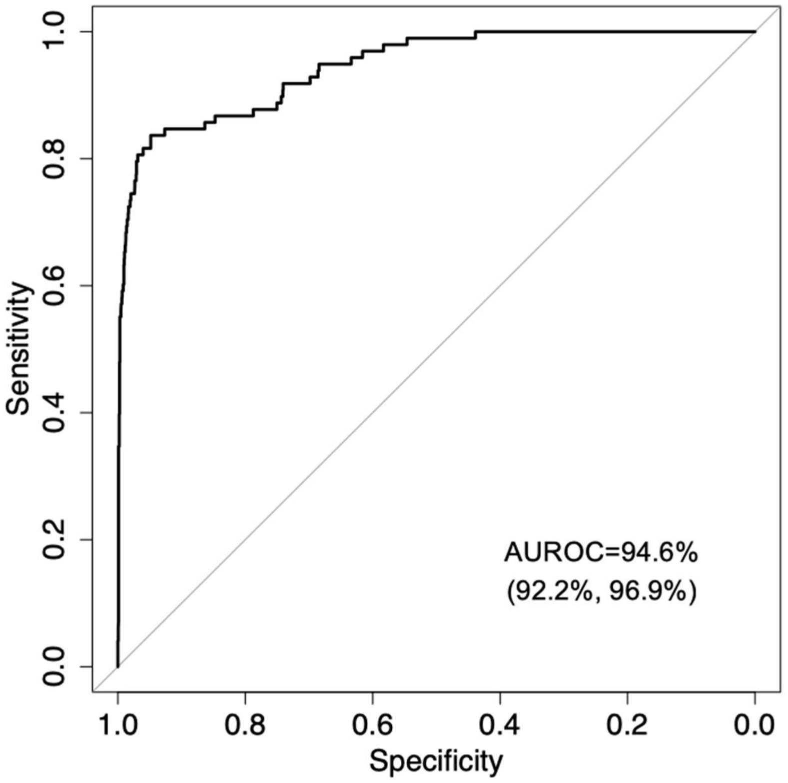

Suppl. Fig. 3: Flowchart illustrating the selection of the Post hoc EURINE-ACT Analysis Cohort from the Final EURINE-ACT Analysis Cohort. For this purpose, we excluded all ACCs that were readily identifiable as ACC either by steroid pattern (mixed or aberrant excess steroid excess) or clinical presentation (large adrenal mass with extra-adrenal metastases), and all ACAs identifiable as benign due to presentation with bilateral macronodular adrenal hyperplasia and isolated cortisol excess.

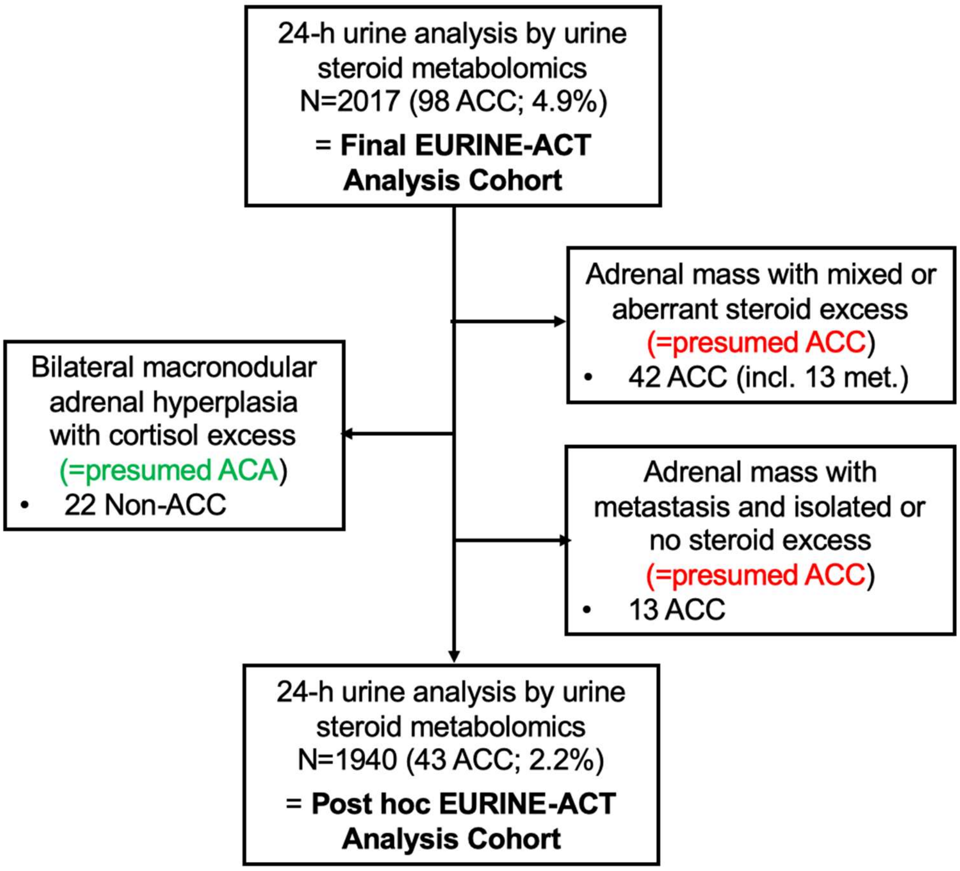

Suppl. Fig. 4: Results of multi-class GMLVQ systems trained and applied to prospective urine steroid metabolome data for the differentiation of adrenocortical carcinoma (ACC) and the three Non-ACC classes. The GMVLQ algorithm prospectively validated in the EURINE-ACT study had been trained to differentiate ACC from ACA,. However, due to the large number of patients recruited in the prospective study, its population also comprised other benign (OB) and other malignant (OM) adrenal masses. Therefore, we trained additional GMVLQ algorithms to see if this would achieve further differentiation. For the results in **Panel A**, a GMLVQ system was obtained by training on a class-balanced randomised set of 260 samples. We display the projections of a particular training set on the two leading eigenvalues of the resulting relevance matrix **Λ.** The corresponding prototype vectors are displayed as large symbols, small filled circles represent the steroid metabolome of individual patients, with each steroid metabolome comprising 15 distinct urinary steroid metabolites measured by LC-MS/MS. The confusion matrix below the graph summarises the average percentages of mis-classifications over 50 training randomly selected training sets. This shows that ACC samples clearly separate from all other tumour groups, while the three non-ACC groups display significant overlap. In **Panel B**, analogous results are shown for a three-class GMLVQ system trained from reduced data sets comprising only ACA, OM and OB tumours. The separation of the three classes appears slightly better than in the four-class setup of Panel A, but still displays significant overlap.

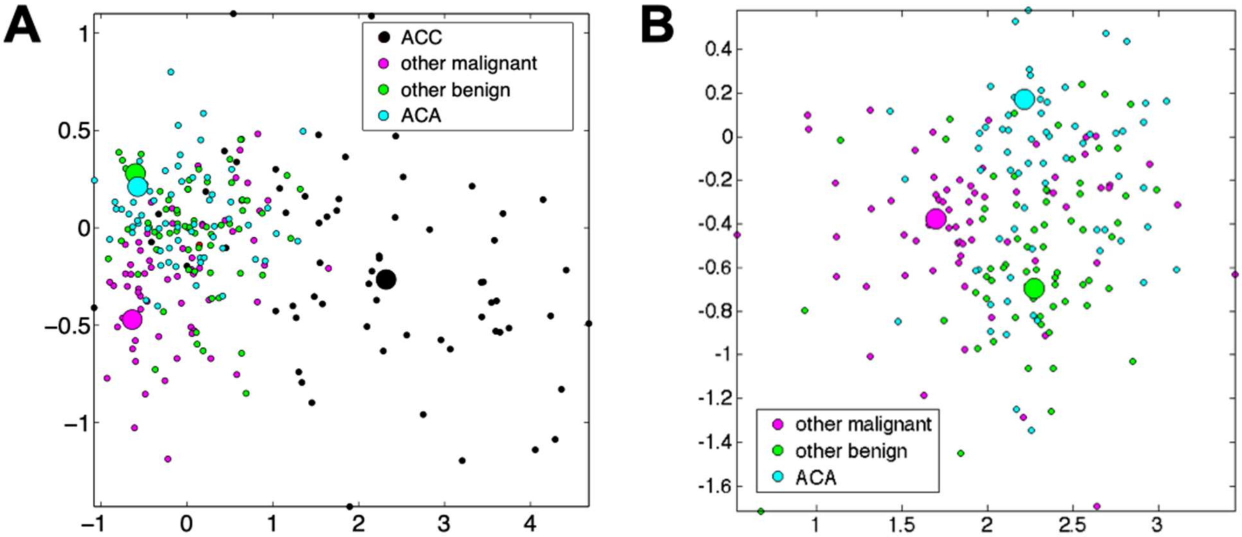

A — Percentage classified as

True Class	ACC	Other malignant	Other benign	ACA
ACC	80.1	2.7	12.0	5.2
Other malign.	4.3	37.4	38.7	19.6
Other benign	3.4	20.0	52.6	24.0
ACA	4.6	21.2	33.6	40.6

B — Percentage classified as

True Class	Other malignant	Other benign	ACA
Other malign.	43.8	36.7	19.5
Other benign	24.8	49.8	25.4
ACA	24.8	33.5	41.7

Supplementary Tables

Suppl. Table 1: Imaging test combinations performed in the EURINE-ACT participants (N=2017) prior to clinical decision on whether the adrenal mass was regarded as benign or malignant. Imaging modalities were carried out according to standard guidelines and included non-contrast computed tomography (CT; (N=1549) with measurement of attenuation in homogeneous tumours, magnetic resonance imaging (MRI; N=342) with chemical shift analysis, fluordeoxyglucose-positron emission tomography (FDG-PET; N=161) as well as CT contrast washout studies (N=21) and follow-up CT (N=664). 37 patients did not undergo additional imaging after the initial contrast CT that had led to the discovery of a large adrenal mass; histopathology confirmed 32 ACA and 5 other benign tumours.

Non-contrast CT	MRI	FDG-PET	CT Contrast Washout	Follow-up CT at 6 months	Contrast CT only + histology	Frequency	%
X	X	X		X		11	0.5
X	X	X				8	0.4
X	X			X		41	2.0
X	X					55	2.7
X		X		X		35	1.7
X		X				58	2.9
X			X			15	0.7
X				X		360	17.8
X						966	47.9
	X	X		X		3	0.1
	X	X				3	0.1
	X			X		37	1.8
	X					184	9.1
		X		X		22	1.1
		X				21	1.0
			X			6	0.3
				X		155	7.7
					X	37	1.8

Suppl. Table 2: Nomenclature and origin of the 15 urinary steroid metabolites quantified by the multi-steroid profiling assay utilizing liquid chromatography-tandem mass spectrometry (LC-MS/MS; single run of 7 minutes duration). * indicates steroids comprised in the "malignant steroid fingerprint" indicative of ACC identified by machine learning in our previous retrospective proof-of-principle study (10).

**Abbreviation**	**Common name**	**Chemical name**	**Metabolite of**
An	Androsterone	5α-androstan-3α-ol-17-one	Androstenedione, testosterone, 5α-dihydrotestosterone
Etio*	Etiocholanolone	5β-androstan-3α-ol-17-one	Androstenedione, testosterone
11β-OH-An	11β-hydroxyandrosterone	5α-androstan-3α,11β -diol-17-one	11β-hydroxy-androstenedione
DHEA	Dehydroepiandrosterone	5-androsten-3β-ol-17-one	DHEA, DHEAS
5-PT*	Pregnenetriol	5-pregnene-3β,17-20α-triol	17-hydroxy-pregnenolone
5-PD*	Pregnenediol	5-pregnene-3β,20α-diol	Pregnenolone
PD*	Pregnanediol	5β-pregnane-3α,20α-diol	Progesterone
17HP*	17-hydroxypregnanolone	5β-pregnane-3α,17α-diol-20-one	17-hydroxy-progesterone
PT*	Pregnanetriol	5β-pregnane-3α,17α,20α-triol	17-hydroxy-progesterone
THS*	Tetrahydro-11-deoxycortisol	5β-pregnane-3α,17α,21-triol-20-one	11-deoxycortisol
F	Cortisol	4-pregnene-11β,17,21-triol-3,20-dione	Cortisol
11β-OHEtio	11β-hydroxyetiocholanolone	5β-androstan-3α,11β-diol-17-one	Cortisol
E	Cortisone	4-pregnene-17α,21-diol-3,11,20-trione	Cortisone
THE	Tetrahydrocortisone	5β-pregnene-3α,17,21-triol-11,20-dione	Cortisone
β-cortolone	β-cortolone	5β-pregnane-3α,17,20β,21-tetrol-11-one	Cortisone

Supp. Table 3: Validation data of the LC-MS/MS multi-steroid profiling assay including limit of detection (LOD), limit of quantitation (LOD), reproductivility, accuracy and low (L), medium (M) and high (H) concentrations; precision, matrix effects, and absolute recovery.

Low, medium and high concentrations; cortisol, cortisone, β-cortolone, 11βOHEt, 11βOHAn, 50, 500 and 500ng/mL; THE, THS, Etio, An 250, 500, 5000ng/mL; 5-PT 10, 100, 500ng/mL; DHEA, 10, 100, 250ng/mL; 5PD 10, 50, 500ng/mL; 17HP 50, 100, 250ng/mL; PT 100, 500, 5000ng/mL; PD 50, 100, 500ng/mL. RSD, relative standard deviation = (standard deviation*100)/average.

Steroid	LOD (ng/mL)	LOQ[a] (µg/24hr)	Reproducibility (RSD%)	Accuracy (RSD %)			Precision (RSD %)			Matrix Effects (%)	Absolute Recovery (%)
				L	M	H	L	M	H		
E	0.3	20	16	6.5	5.6	3.1	1.9	2.7	1.6	-0.003	99
F	0.7	20	6	8.3	3.0	2.3	4.8	2.6	1.0	-0.003	102
THE	5.2	30	5	11	12	4.4	2.2	2.0	3.0	0.03	103
β-cortolone	1.6	20	5	14	4.0	6.0	14	7.0	5.4	0.007	102
11βOHEt	56.8	96	12	18	5.0	7.0	17	5.0	7.0	8.0^{-04}	108
11βOHAn	30.6	105	14	18	5.8	8.3	13	10	6.0	-9.0^{-04}	122
5PT	2.4	43	6	19	12	3.9	7.9	12	5.5	0.003	94
DHEA	3.4	22	22	9.0	10	1.6	13	17	7.5	0.0007	96
THS	31.4	55	18	2.1	2.5	4.7	5.3	6.9	4.4	-0.002	96
Etio	5.4	10	4	3.5	3.3	3.7	4.4	5.0	3.6	0.02	102
5PD	8.1	55	10	29	16	2.6	14	11	5.3	-0.003	74
An	2.7	13	6	3.0	3.0	4.2	5.8	4.7	5.1	0.008	100
17HP	1.6	24	12	15	9.2	3.8	15	13	0.4	8.0^{-05}	96
PT	1.4	110	6	6.3	3.2	4.3	6.1	7.3	4.6	0.004	96
PD	15.2	88	13	21	18	8.5	23	16	11	0.004	85

[a]based on an average signal-to-noise ratio 10:1 in patient samples

Suppl. Table 4: Characteristics of centre-specific patient recruitment during the 66 months of prospective recruitment to ENSAT EURINE-ACT. The table lists the centre-specific recruitment periods, the number of patients recruited per centre and number and prevalence of adrenocortical carcinoma (ACC) cases for each of the 21 clinical centres that agreed to participate in EURINE-ACT. On this basis, centres 1-14 (total recruitment N=2068) were classified as non-selective recruitment centres while the remaining seven centres 15-21 (total recruitment N=101) were classified as selective. Centres with selective enrolment did not pursue consecutive enrolment as requested by the protocol, but prioritized enrolment of patients with large and indeterminate tumours, thus resulting in a high proportion of ACC not reflective of routine caseload. Consent rate was >95% of all patients approached in each of participating centres.

Centre No	Recruitment period (months)	Number of eligible patients consented (mean N/year)	Number of ACC patients (% of total n)	Centre Recruitment Classification
1	36	278 (93)	15 (5.4)	Non-selective
2	60	264 (53)	15 (5.7)	Non-selective
3	48	246 (62)	4 (1.6)	Non-selective
4	48	234 (59)	7 (3.0)	Non-selective
5	60	225 (45)	9 (4.0)	Non-selective
6*	60	158 (32)	21 (13.3)	Non-selective
7*	48	153 (38)	14 (9.2)	Non-selective
8	54	132 (29)	5 (3.8)	Non-selective
9	36	93 (31)	1 (1.1)	Non-selective
10*	36	73 (24)	8 (11.0)	Non-selective
11	24	69 (35)	0 (0)	Non-selective
12	24	52 (26)	1 (1.9)	Non-selective
13	30	51 (20)	3 (5.9)	Non-selective
14	24	40 (20)	0 (0)	Non-selective
15	36	24 (8.0)	5 (20.8)	Selective
16	36	20 (6.7)	2 (10.0)	Selective
17	30	19 (7.6)	6 (31.6)	Selective
18	36	17 (5.7)	8 (53.0)	Selective
19	30	17 (6.8)	6 (35.0)	Selective
20	48	3 (0.75)	3 (100%)	Selective
21	48	1 (0.25)	1 (100%)	Selective

* One of three major ACC specialist centres in Europe, thus, higher ACC rate than other centres

Suppl. Table 5: Distribution of Pathologies in the EURINE-ACT cohort. Underlying pathologies are listed as N (%) for the whole cohort and for incidentally and non-incidentally discovered tumours, respectively, as well as for those adrenal tumours that underwent surgical removal (adrenalectomy group).

	**All participants**<br><br>**Number**<br>**(% of total)**<br>(% of group)	**Non-incidentally discovered tumours**<br><br>**Number**<br>**(% of total)**<br>(% of group)	**Incidentally discovered tumours**<br><br>**Number**<br>**(% of total**<br>(% of group)	**Adrenal masses surgically removed (Adrenalectomy Group)**<br><br>**Number**<br>**(% of total)**<br>(% of group)
**Total number**	**2017 (100)**	**331 (16.4)**	**1686 (83.6)**	**563 (27.9)**
**Adrenocortical carcinoma (ACC)**	**98 (4.9)**	**55 (16.6)**	**43 (2.6)**	**84 (14.9)**
**Benign adrenocortical adenomas (ACA)**	**1767 (87.6)**	**254 (76.7)**	**1513 (89.7)**	**370 (65.7)**
Non-functioning adenoma	913 (51.7)	71 (28.0)	842 (55.7)	81 (21.9)
Mild autonomous cortisol secretion	602 (34.1)	30 (11.8)	572 (37.8)	105 (28.4)
Aldosterone-producing adenoma	153 (8.7)	118 (46.5)	35 (2.3)	119 (32.2)
Cortisol-producing adenoma	77 (4.4)	31 (12.2)	46 (3.0)	51 (13.8)
Bilateral macronodular hyperplasia with cortisol excess	22 (1.2)	4 (1.6)	18 (1.2)	14 (3.8)
**Other malignant (OM) masses**	**65 (3.2)**	**9 (2.7)**	**56 (3.3)**	**50 (8.9)**
Metastasis	39 (60.0)	9 (100.0)	30 (53.6)	30 (60.0)
Primary Adrenal Lymphoma	8 (12.3)	0 (0.0)	8 (14.3)	4 (8.0)
Leiomyosarcoma	5 (7.7)	0 (0.0)	5 (8.9)	4 (8.0)
Angiosarcoma	4 (6.2)	0 (0.0)	4 (7.1)	4 (8.0)
Liposarcoma	4 (6.2)	0 (0.0)	4 (7.1)	3 (6.0)
Neuroblastoma	2 (3.1)	0 (0.0)	2 (3.6)	2 (4.0)
Sarcoma	2 (3.1)	0 (0.0)	2 (3.6)	2 (4.0)
Castleman	1 (1.5)	0 (0.0)	1 (1.8)	1 (2.0)
**Other benign (OB) masses**	**87 (4.3)**	**13 (3.9)**	**74 (4.4)**	**59 (10.5)**
Myelolipoma	28 (32.2)	3 (23.1)	25 (33.8)	17 (28.8)
Cyst	17 (19.5)	2 (15.4)	15 (20.3)	12 (20.3)
Pheochromocytoma	10 (11.5)	1 (7.7)	9 (12.2)	8 (13.6)
Ganglioneuroma	8 (9.2)	3 (23.1)	5 (6.8)	8 (13.6)
Hemangioma	8 (9.2)	1 (7.7)	7 (9.5)	7 (11.9)
Hematoma	8 (9.2)	1 (7.7)	7 (9.5)	2 (3.4)
Schwannoma	2 (2.3)	1 (7.7)	1 (1.4)	2 (3.4)
Lymphangioma	2 (2.3)	0 (0.0)	2 (2.7)	1 (1.7)
Hepatic adenoma	1 (1.1)	1 (7.7)	0 (0.0)	0 (0.0)
Pseudocyst	1 (1.1)	0 (0.0)	1 (1.4)	1 (1.7)
Stromal tumour	1 (1.1)	0 (0.0)	1 (1.4)	1 (1.7)
Angiolipoma	1 (1.1)	0 (0.0)	1 (1.4)	0 (0.0)

Suppl. Table 6: Reference standards applied to the adrenal masses in the EURINE-ACT participants (N=2017).

	Adreno-cortical adenomas (ACA)	Adreno-cortical carcinomas (ACC)	Other malignant (OM) masses	Other benign (OB) masses
Total, n	1,767	98	65	87
Histopathology, n (%)	370 (21%)	91 (93%)*	65 (100%)*	59 (68%)
No histopathology, n (%)	1397 (79%)	7 (7%)**	0 (0%)	28 (32%)
Number of patients with imaging follow up > 6 months, n (%)	577 (33%)	0 (0%)	0 (0%)	16 (18%)
Duration (months) of imaging follow-up, median (IQR)	17 (10, 72)	N/A	N/A	19 (10, 92)
Number of patients with clinical follow# up only > 12 months, n (%)	820 (46%)	0 (0%)	0 (0%)	12 (14%)
Duration (months) of clinical follow-up#, median (IQR)	24 (12, 26)	N/A	N/A	N/A***

* Seven ACC patients and 15 patients with OM masses underwent biopsy only of the adrenal mass (no adrenalectomy). Within the OM group, biopsy revaled metastasis of non-adrenal primary tumours (N=9), primary adrenal lymphoma (N=4), liposarcoma (N=1), and leiomyosarcoma (N=1).

** Seven ACC patients presented with a large adrenal tumour, metastases and steroid hormone excess, indicative of ACC, with no feasible alternative diagnosis, which allows for the diagnosis of ACC without histopathology[13].

*** Twelve benign adrenal myelolipomas were unequivocally diagnosed by their characteristic imaging findings[7] and, therefore, did not undergo further follow-up.

clinical follow up was defined as a face-to-face visit including a physical examination.

Suppl. Table 7: Sensitivity and specificity (%) when using different cut-offs for maximum tumour diameter (N=2017) and tumour attenuation measured in Hounsfield Units (HU) or tumour heterogeneity on unenhanced computed tomography (CT; N=1549). *one-sided 97.5% Confidence Interval (CI).

Criteria for positive test result	True Positive/ACC; Sensitivity % (95% CI)	True Negative/Non-ACC; Specificity % (95% CI)
**Tumour diameter (N=2017)**		
≥2cm	98/98; 100.0 (96.3, 100.0)*	576/1919; 30.0 (28.0, 32.1)
≥4cm	96/98; 98.0 (92.8, 99.8)	1527/1919; 79.6 (77.7, 81.3)
≥6cm	82/98; 83.7 (74.8, 90.4)	1793/1919; 93.4 (92.2, 94.5)
**Unenhanced CT (N=1549)**		
HU≥10 & not heterogeneous	33/98; 33.7 (24.4, 43.9)	950/1451; 65.5 (63.0, 67.9)
HU>20 & not heterogeneous	32/98; 32.7 (23.5, 42.9)	1183/1451; 81.5 (79.4, 83.5)
Heterogeneous	65/98; 66.3 (56.1, 75.6)	1429/1451; 98.5 (97.7, 99.0)
HU≥10 or heterogeneous	98/98; 100.0 (96.3, 100.0)*	928/1451; 64.0 (61.4, 66.4)
HU>20 or heterogeneous	97/98; 99.0 (94.4, 100.0)	1161/1451; 80.0 (77.9, 82.0)

Suppl. Table 8: Cross tabulations of the results of urine steroid metabolomics (USM) risk score classification (low, moderate, or high risk of adrenocortical carcinoma [ACC]) and imaging characteristics by tumour diameter and underlying diagnosis (Non-ACC [N=1919] vs. ACC [N=98]).

Non-ACC – Tumour diameter <4cm – USM

Imaging characteristics	Low Risk	Moderate Risk	High Risk	Total
Negative	762	428	93	1283
Positive	129	97	18	244
Total	891	525	111	1527

ACC – Tumour diameter <4cm – USM

Imaging characteristics	Low Risk	Moderate Risk	High Risk	Total
Negative	0	0	0	0
Positive	0	1	1	2
Total	0	1	1	2

Non-ACC – Tumour diameter ≥4cm – USM

Imaging characteristics	Low Risk	Moderate Risk	High Risk	Total
Negative	147	72	21	240
Positive	69	58	25	152
Total	216	130	46	392

ACC – Tumour diameter ≥4cm – USM

Imaging characteristics	Low Risk	Moderate Risk	High Risk	Total
Negative	0	0	1	1
Positive	2	12	81	95
Total	2	12	82	96

Suppl. Table 9: Cross tabulations of the results of urine steroid metabolomics (USM) risk score classification (low, moderate, or high risk of adrenocortical carcinoma [ACC]) and imaging characteristics on CT (unenhanced tumour attenuation >20HU or tumour heterogeneity were considered as indicative of ACC [=postitive]; total N=1549) by tumour diameter and underlying diagnosis (Non-ACC [N=1451] vs. ACC [N=98]).

Non-ACC, Tumour diameter <4cm

	USM			
**Unenhanced CT >20HU or heterogeneity**	Low Risk	Moderate Risk	High Risk	Total
Negative	598	320	63	981
Positive	96	68	12	176
Total	694	388	75	1157

ACC, Tumour diameter <4cm

	USM			
**Unenhanced CT >20HU or heterogeneity**	Low Risk	Moderate Risk	High Risk	Total
Negative	0	0	0	0
Positive	0	1	1	2
Total	0	1	1	2

Non-ACC, Tumour diameter ≥4cm

	USM			
**Unenhanced CT >20HU or heterogeneity**	Low Risk	Moderate Risk	High Risk	Total
Negative	113	54	13	180
Positive	48	47	19	114
Total	161	101	32	294

ACC, Tumour diameter ≥4cm

	USM			
**Unenhanced CT >20HU or heterogeneity**	Low Risk	Moderate Risk	High Risk	Total
Negative	0	0	1	1
Positive	2	12	81	95
Total	2	12	82	96

Suppl. Table 10: Cross tabulations of the results of urine steroid metabolomics (USM) risk score classification (low, moderate, or high risk of adrenocortical carcinoma [ACC]) and imaging with MRI chemical shift analysis (total N=342) by tumour diameter and underlying diagnosis (Non-ACC [N=341] vs. ACC [N=1]).

Non-ACC – Tumour diameter <4cm

**MRI**	USM Low Risk	USM Moderate Risk	USM High Risk	Total
Negative	115	75	28	218
Positive	27	24	5	56
Total	142	99	33	274

ACC – Tumour diameter <4cm

**MRI**	USM Low Risk	USM Moderate Risk	USM High Risk	Total
Negative	0	0	0	0
Positive	0	0	0	0
Total	0	0	0	0

Non-ACC – Tumour diameter ≥4cm

**MRI**	USM Low Risk	USM Moderate Risk	USM High Risk	Total
Negative	20	9	6	35
Positive	18	9	5	32
Total	38	18	11	67

ACC – Tumour diameter ≥4cm

**MRI**	USM Low Risk	USM Moderate Risk	USM High Risk	Total
Negative	0	0	0	0
Positive	0	0	1	1
Total	0	0	1	1

Suppl. Table 11: Cross tabulations of the results of urine steroid metabolomics (USM) risk score classification (low, moderate, or high risk of adrenocortical carcinoma [ACC]) and imaging by FDG-PET (total N=161) by tumour diameter and underlying diagnosis (Non-ACC [N=159] vs. ACC [N=2]).

Non-ACC
Tumour diameter <4cm
USM

**PET**	Low Risk	Moderate Risk	High Risk	Total
Negative	55	32	10	97
Positive	6	7	0	13
Total	61	39	10	110

ACC
Tumour diameter <4cm
USM

**PET**	Low Risk	Moderate Risk	High Risk	Total
Negative	0	0	0	0
Positive	0	0	0	0
Total	0	0	0	0

Non-ACC
Tumour diameter ≥4cm
USM

**PET**	Low Risk	Moderate Risk	High Risk	Total
Negative	23	10	3	36
Positive	4	7	2	13
Total	27	17	5	49

ACC
Tumour diameter ≥4cm
USM

**PET**	Low Risk	Moderate Risk	High Risk	Total
Negative	0	0	0	0
Positive	0	0	2	2
Total	0	0	2	2

Suppl. Table 12: Intermediate testing steps for strategies combining the three index tests (tumour diameter, imaging characteristics, and urine steroid metabolomics [USM]). Measures reported with 95% confidence intervals. USM urine steroid metabolomics; OB Other Benign; OM Other Malignant; shaded areas show results for Non-ACC results by group; †Sensitivity; ‡Specificity.

	ACC	Non ACC	ACA	OB	OM	Total	**% of ACC cases**	**% of non-ACC cases**	**Likelihood Ratio**	**Post-test probability of ACC (per 100)**
**Double test strategy: Tumour diameter AND Imaging characteristics**										
**Results of second test (Imaging characteristics)** for participants with Tumour diameter ≥4cm (N=488)										
Tumour ≥4cm AND **Imaging characteristics positive**	95	152	83	24	45	247	†99.0 (94.3, 100.0)	38.8 (33.9, 43.8)	2.6 (2.3, 2.9)	38.5 (32.4, 44.8)
Tumour ≥4cm AND **Imaging characteristics negative**	1	240	213	26	1	241	1.0 (0.0, 5.7)	‡61.2 (56.2, 66.1)	0.02 (0.00, 0.12)	0.4 (0.0, 2.3)
Total	96	392	296	50	46	**488**				
**Double test strategy: Tumour diameter AND Urine Steroid Metabolomics (USM)**										
**Results of second test (USM)** for participants with Tumour diameter ≥4cm (N=488)										
Tumour ≥4cm AND **USM High Risk (HR) score**	82	46	33	6	7	128	85.4 (76.7, 91.8)	11.7 (8.7, 15.3)	7.3 (5.5, 9.7)	64.1 (55.1, 72.3)
Tumour ≥4cm AND **USM Moderate Risk (MR) score**	12	130	85	25	20	142	12.5 (6.6, 20.8)	33.2 (28.5, 38.1)	0.38 (0.22, 0.65)	8.5 (4.4, 14.3)
Tumour ≥4cm AND **USM Low Risk (LR) score**	2	216	178	19	19	218	2.1 (0.3, 7.3)	55.1 (50.0, 60.1)	0.04 (0.01, 0.15)	0.9 (0.1, 3.3)
Total	96	392	296	50	46	**488**				
**Double test strategy: Urine steroid metabolomics (USM) AND Imaging characteristics**										
**Result of second test (Imaging characteristics)** for participants with USM-HR or -MR (N=908)										
USM-HR AND **Imaging characteritics positive**	82	43	35	2	6	125	85.4 (76.7, 91.8)	5.3 (3.9, 7.1)	16.1 (11.9, 21.8)	65.6 (56.6, 73.9)
USM-MR AND **Imaging characteristics positive**	13	155	97	30	28	168	13.5 (7.4, 22.0)	19.1 (16.4, 22.0)	0.71 (0.42, 1.20)	7.7 (4.2, 12.9)
USM-HR/-MR AND **Imaging characteristics negative**	1	614	589	24	1	615	1.0 (0.0, 5.7)	75.6 (72.5, 78.5)	0.01 (0.00, 0.10)	0.1 (0.0, 0.9)
Total	96	812	721	56	35	**908**				
**Result of second test (USM)** for participants with positive Imaging characteristics (N=493)										
Imaging positive AND **USM-HR**	82	43	35	2	6	125	84.5 (75.8, 91.1)	10.9 (8.0, 14.3)	7.8 (5.8, 10.5)	65.6 (56.6, 73.9)
Imaging positive AND **USM-MR**	13	155	97	30	28	168	13.4 (7.3, 21.8)	39.1 (34.3, 44.1)	0.34 (0.20, 0.58)	7.7 (4.2, 12.9)
Imaging positive AND **USM-LR**	2	198	157	12	29	200	2.1 (0.3, 7.3)	0.34 (0.01, 0.16)	0.04 (0.01, 0.16)	1.0 (0.1, 3.6)
Total	97	396	289	44	63	**493**				
**Triple test strategy: Tumour diameter AND USM test AND Imaging characteristics**										
**Result of third test (Imaging characteristics)** for participants with Tumour diameter ≥4cm AND USM-HR or -MR (N=270)										
Tumour ≥4cm AND USM-HR AND **Imaging positive**	81	25	17	2	6	106	86.2 (77.5, 92.4)	14.2 (9.4, 20.3)	6.1 (4.2, 8.8)	76.4 (67.2, 84.1)
Tumour ≥4cm AND USM-MR AND **Imaging positive**	12	58	23	15	20	70	12.8 (6.8, 21.2)	33.0 (26.1, 40.4)	0.39 (0.22, 0.68)	17.1 (9.2, 28.0)
Tumour ≥4cm AND USM-HR/-MR AND **Imaging negative**	1	93	78	14	1	94	1.1 (0.0, 5.8)	52.8 (45.2, 60.4)	0.02 (0.00, 0.14)	1.1 (0.0, 5.8)
Total	94	176	118	31	27	**270**				
**Result of third test (USM)** for participants with Tumour diameter ≥4cm AND positive Imaging characteristics (N=247)										
Tumour ≥4cm AND Imaging positive AND **USM-HR**	81	25	17	2	6	106	85.3 (76.5, 91.7)	16.4 (10.9, 23.3)	5.2 (3.6, 7.5)	76.4 (67.2, 84.1)
Tumour ≥4cm AND Imaging positive AND **USM-MR**	12	58	23	15	20	70	12.6 (6.7, 21.0)	38.2 (30.4, 46.4)	0.33 (0.19, 0.58)	17.1 (9.2, 28.0)
Tumour ≥4cm AND Imaging positive AND **USM-LR**	2	69	43	7	19	71	2.1 (0.3, 7.4)	45.4 (37.3, 53.7)	0.05 (0.01, 0.18)	2.8 (0.3, 9.8)
Total	95	152	83	24	45	**247**				

Suppl. Table 13: Tumour-related steroid secretion patterns according to clinical presentation and results of routine serum biochemistry in the EURINE-ACT participants. We compared ACC vs. Non-ACC sub-divided by maximum tumour diameter (< or ≥6cm). Data are presented as N (%). Twenty-six ACCs (26.5%) were metastatic upon first diagnosis of the adrenal mass, numbers of metastatic tumours indicated per steroid category.

**Tumour-related steroid secretion pattern**	**ACC <6cm (N=22)**	**ACC ≥6cm (N=76)**	**Non-ACC <6cm (N=1808)**	**Non-ACC ≥6cm (N=111)**
**Clinically overt and biochemical signs of steroid excess**				
Mixed (or aberrant) steroid excess	4 (22.7%)	24 (34.2%)<br>12/24 metastatic	0 (0%)	0 (0%)
Isolated cortisol excess	2 (9.1%)<br>1/2 metastatic	10 (13.2%)<br>6/10 metastatic	74 (4.1%)	3 (2.7%)
Isolated aldosterone excess	0 (0%)	0 (0%)	153 (8.5%)	0 (0%)
Isolated androgen excess	2 (9.1%)	0 (0%)	0 (0%)	0 (0%)
**No clinical but biochemical signs of steroid excess**				
Mixed (or aberrant) steroid excess	4 (18.2%)	10 (13.2%)<br>1/10 metastatic	0 (0%)	0 (0%)
Isolated cortisol excess	4 (18.2%)	4 (5.3%)	604 (33.4%)*	20 (18.0%)*
Isolated aldosterone excess	0 (0%)	1 (1.3%)	0 (0%)	0 (0%)
Isolated androgen excess	1 (4.6%)	7 (9.2%)<br>2/7 metastatic	0 (0%)	0 (0%)
**Neither clinical nor biochemical signs of steroid excess**				
No steroid excess	4 (18.2%)	18 (23.7%)<br>4/18 metastatic	978 (54.1%)	87 (8.4%)

* In the Non-ACC category, 21 participants with maximum tumour diameter <6cm (1.2%) and one patient ≥6cm (0.9%) presented with bilateral macronodular adrenal hyperplasia and biochemical cortisol excess; these are included in the overall numbers in the respective category.

Suppl. Table 14: Post hoc sensitivity analysis. Performance of tests and test strategies in the EURINE-ACT cohort (N=1940) after exclusion of patients identifiable as ACC or ACA by steroid pattern or clinical presentation. Measures reported with 95% confidence intervals.
USM, urine steroid metabolomics; ACC, adrenocortical carcinoma; ACA, adrenocortical adenoma; OB, Other Benign; OM, Other Malignant; the shaded areas show results for the Non-ACC sub-groups ACA, OB, and OM; †Sensitivity; ‡Specificity.

		ACC (43)	Non ACC (1897)	ACA (1745)	OB (87)	OM (65)	Total (1940)	% of ACC cases	% of Non-ACC cases	Likelihood Ratio (LR)	Post-test probability of ACC (per 100)
**SINGLE TEST STRATEGIES**											
**Single test: Tumour Diameter**											
Positive	≥4cm	42	383	287	50	46	425	†97.7 (87.7, 99.9)	20.2 (18.4, 22.1)	4.8 (4.4, 5.4)	9.9 (7.2, 13.1)
Negative	<4cm	1	1514	1458	37	19	1515	2.3 (0.1, 12.3)	‡79.8 (77.9, 81.6)	0.03 (0.01, 0.20)	0.1 (0.0, 0.4)
**Single test: Imaging characteristics**											
Positive	Positive	42	394	287	44	63	436	†97.7 (87.7, 99.9)	20.8 (19.0, 22.7)	4.7 (4.3, 5.2)	9.6 (7.0, 12.8)
Negative	Negative	1	1503	1458	43	2	1504	2.3 (0.1, 12.3)	‡79.2 (77.3, 81.0)	0.03 (0.00, 0.20)	0.1 (0.0, 0.4)
**Single test: Urine Steroid Metabolomics (USM)**											
High	High Risk of ACC (USM-HR)	33	155	141	7	7	188	76.7 (61.4, 88.2)	8.2 (7.0, 9.5)	9.4 (7.5, 11.7)	17.6 (12.4, 23.8)
Moderate	Moderate Risk of ACC (USM-HR)	10	648	571	49	28	658	23.3 (11.8, 38.6)	34.2 (32.0, 36.3)	0.7 (0.4, 1.2)	1.5 (0.7, 2.8)
Low	Low Risk of ACC (USM-LR)	2	1094	1033	31	30	1094	0.0 (0.0, 8.2)*	57.7 (55.4, 59.9)	-	0.0 (0.0, 0.4)*
**COMBINED TEST STRATEGIES**											
**Double test strategy: Tumour Diameter AND Imaging characteristics**											
Positive	Tumour diameter ≥4cm AND Imaging characteristics positive	41	151	82	24	45	192	†95.3 (84.2, 99.4)	8.0 (6.8, 9.3)	12.0 (10.1, 14.2)	21.4 (15.8, 27.8)
Negative	Tumour diameter <4cm AND/OR Imaging characteristics negative	2	1746	1663	63	20	1748	4.7 (0.6, 15.8)	‡92.0 (90.7, 93.2)	0.05 (0.01, 0.20)	0.1 (0.0, 0.4)
**Double test strategy: Tumour Diameter AND Urine Steroid Metabolomics (USM)**											
High	Tumour diameter ≥4cm AND USM high risk of ACC (USM-HR)	33	45	32	6	7	78	76.7 (61.4, 88.2)	2.4 (1.7, 3.2)	32.4 (23.2, 45.1)	42.3 (31.2, 54.0)
Moderate	Tumour diameter ≥4cm AND USM moderate risk of ACC (USM-MR)	9	128	83	25	20	137	20.9 (10.0, 36.0)	6.7 (5.7, 8.0)	3.1 (1.7, 5.7)	6.6 (3.1, 12.1)
Low	Tumour diameter <4cm AND/OR USM low risk of ACC (USM-LR)	1	1724	1630	56	38	1725	2.3 (0.1, 12.3)	90.9 (89.5, 92.1)	0.03 (0.02, 0.18)	0.1 (0.0, 0.3)
**Double test strategy: Imaging characteristics AND Urine Steroid Metabolomics (USM)**											
High	Imaging characteristics positive AND USM high risk of ACC (USM-HR)	32	42	34	2	6	74	74.4 (58.8, 86.5)	2.2 (1.6, 3.0)	33.6 (23.8, 47.5)	43.2 (31.8, 55.3)

Moderate	Imaging characteristics positive AND USM moderate risk of ACC (USM-MR)	10	154	96	30	28	164	23.3 (11.8, 38.6)	8.1 (6.9, 9.4)	2.9 (1.6, 5.0)	6.1 (0.0, 0.3)
Low	Imaging characteristics negative AND/OR USM low risk of ACC (USM-LR)	1	1701	1615	55	31	1702	2.3 (0.1, 12.3)	89.7 (88.2, 91.0)	0.03 (0.01, 0.18)	0.1 (0.0, 0.3)
**Triple test strategy: Tumour Diameter AND USM test AND Imaging characteristics**											
High	Tumour diameter ≥4cm AND USM high risk of ACC (USM-HR) AND Imaging characteristics positive	32	25	17	2	6	57	74.4 (58.8, 86.5)	1.3 (0.9, 1.9)	56.5 (36.8, 86.5)	56.1 (42.4, 69.3)
Moderate	Tumour diameter ≥4cm AND USM moderate risk of ACC (USM-MR) AND Imaging characteristics positive	9	57	22	15	20	66	20.9 (10.0, 36.0)	3.0 (2.3, 3.9)	7.0 (3.7, 13.1)	13.6 (6.4, 24.3)
Low	Tumour diameter <4cm AND/OR USM low AND/OR Imaging characteristics negative	2	1815	1706	70	39	1817	4.7 (0.6, 15.8)	95.7 (94.7, 96.5)	0.05 (0.01, 0.19)	0.1 (0.0, 0.4)

Suppl. Table 15: Performance of tests and test strategies when detecting malignant adrenal masses other than adrenocortical carcinoma (other malignant; OM). Measures reported with 95% confidence intervals. USM, urine steroid metabolomics; OM, Other Malignant; OB, Other Benign; ACA, adrenocortical adenoma; ACC, adrenocortical carcinoma; Non-OM =ACA+OB+ACC; shaded areas show results for Non-OM results by group; †Sensitivity; ‡Specificity.

		OM (65)	Non-OM (1952)	ACA (1767)	OB (87)	ACC (98)	Total	% of OM cases	% of non-OM cases	Likelihood Ratio	Post-test probability of OM (per 100)
**Single test strategy: Tumour Diameter**											
Positive	≥4cm	46	442	296	50	96	488	70.8 (58.2, 81.4)	22.6 (20.8, 24.6)	3.1 (2.6, 3.7)	9.4 (7.0, 12.4)
Negative	<4cm	19	1510	1471	37	2	1529	29.2 (18.6, 41.8)	77.4 (75.4, 79.2)	0.38 (0.26, 0.55)	1.2 (0.7, 1.9)
**Single test strategy: Imaging characteristics**											
Positive	Imaging characteristics positive	63	430	289	44	97	493	96.9 (89.3, 99.7)	22.0 (20.2, 23.9)	4.4 (4.0, 4.8)	12.8 (10.0, 16.1)
Negative	Imaging characteristics negative	2	1522	1478	43	1	1524	3.1 (0.4, 10.7)	78.0 (76.1, 79.8)	0.04 (0.01, 0.15)	0.1 (0.0, 0.5)
**Single test strategy: Urine steroid metabolomics (USM)**											
High	USM High Risk score (USM-HR)	7	233	143	7	83	240	10.8 (4.4, 20.9)	11.9 (10.5, 13.5)	0.9 (0.44, 1.84)	2.9 (1.2, 5.9)
Moderate	USM Moderate Risk score (USM-MR)	28	640	578	49	13	668	43.1 (30.8, 56.0)	32.8 (30.7, 34.9)	1.31 (0.99, 1.75)	4.2 (2.8, 6.0)
Low	USM Low Risk score (USM-LR)	30	1079	1046	31	2	1109	46.2 (33.7, 59.0)	55.3 (53.0, 57.5)	0.83 (0.64, 1.09)	2.7 (1.8, 3.8)
**Double test strategy: Tumour Diameter AND Imaging characteristics**											
Positive	Tumour ≥4cm AND Imaging characteristics positive	45	202	83	24	95	247	69.2 (56.6, 80.1)	10.3 (9.0, 11.8)	6.7 (5.4, 8.2)	18.2 (13.6, 23.6)
Negative	Tumour <4cm AND/OR Imaging characteristics negative	20	1750	1684	63	3	1770	30.8 (20.0, 43.4)	89.7 (88.2, 91.0)	0.34 (0.24, 0.49)	1.1 (0.7, 1.7)
**Double test strategy: Tumour Diameter AND USM**											
High	Tumour ≥4cm AND USM-HR	7	121	33	6	82	128	10.8 (4.4, 20.9)	6.2 (5.2, 7.4)	1.7 (0.9, 3.6)	5.5 (2.2, 10.9)
Moderate	Tumour ≥4cm AND USM-MR	20	122	85	25	12	142	30.8 (19.9, 43.4)	6.3 (5.2, 7.4)	4.92 (3.29, 7.37)	14.1 (8.8, 20.9)
Low	Tumour <4cm AND/OR USM-LR	38	1709	1649	56	4	1747	58.5 (45.6, 70.6)	87.6 (86.0, 89.0)	0.67 (0.54, 0.82)	2.2 (1.5, 3.0)
**Double test strategy: Imaging characteristics AND USM**											
High	Imaging characteristics positive AND USM high	6	119	35	2	82	125	9.2 (3.5, 19.0)	6.1 (5.1, 7.3)	1.8 (0.9, 3.6)	4.8 (1.9, 10.2)
Moderate	Imaging characteristics positive AND USM moderate	28	140	97	30	13	168	43.1 (30.8, 56.0)	7.2 (6.1, 8.4)	6.01 (4.35, 8.29)	16.7 (11.4, 23.2)
Low	Imaging characteristics negative AND/OR USM low	31	1693	1635	55	3	1724	(47.7 (35.1, 60.5)	86.7 (85.1, 88.2)	0.55 (0.43, 0.71)	1.8 (1.2, 2.5)
**Triple test strategy: Tumour Diameter AND Imaging characteristics AND USM**											
High	Tumour ≥4cm AND Imaging positive AND USM-HR	6	100	17	2	81	106	9.2 (3.5, 19.0)	5.1 (4.2, 6.2)	1.8 (0.8, 4.0)	5.7 (2.1, 11.9)
Moderate	Tumour ≥4cm AND Imaging positive AND USM-M/-L	39	102	66	22	14	141	60.0 (47.1, 72.0)	5.2 (4.3, 6.3)	11.5 (8.7, 15.1)	27.7 (20.5, 35.8)
Low	Tumour <4cm OR Imaging negative AND any USM result	20	1750	1684	63	3	1770	30.8 (19.9, 43.4)	89.7 (88.2, 91.0)	0.34 (0.24, 0.49)	1.1 (0.7, 1.7)

Suppl. Table 16: Cross tabulations of the results of urine steroid metabolomics (USM) risk score classification (low, moderate, or high risk of adrenocortical carcinoma [ACC]) and imaging characteristics by tumour diameter and diagnosis in Non-ACC participants (benign adrenocortical adenoma [ACA; N=1767, other benign [OB; N=87] and other malignant [OM; N=65] adrenal masses.

	**ACA Tumour diameter <4cm USM**				**OB Tumour diameter <4cm USM**				**OM Tumour diameter <4cm USM**			
**Imaging characteristics**	Low Risk	Moderate Risk	High Risk	Total	Low Risk	Moderate Risk	High Risk	Total	Low Risk	Moderate Risk	High Risk	Total
Negative	754	419	92	1265	7	9	1	17	1	0	0	1
Positive	114	74	18	206	5	15	0	20	10	8	0	18
Total	868	493	110	1471	12	24	1	37	11	8	0	19

	**ACA Tumour diameter ≥4cm USM**				**OB Tumour diameter ≥4cm USM**				**OM Tumour diameter ≥4cm USM**			
**Imaging characteristics**	Low Risk	Moderate Risk	High Risk	Total	Low Risk	Moderate Risk	High Risk	Total	Low Risk	Moderate Risk	High Risk	Total
Negative	135	62	16	213	12	10	4	26	0	0	1	1
Positive	43	23	17	83	7	15	2	24	19	20	6	45
Total	178	85	33	296	19	25	6	50	19	20	7	46

Suppl. Table 17: Cross tabulations of the results of urine steroid metabolomics (USM) risk score classification (low, moderate, or high risk of adrenocortical carcinoma [ACC]) and imaging characteristics on unenhanced CT (Total N=1549) by tumour diameter and diagnosis in Non-ACC participants with either benign adrenocortical adenoma [ACA; N=1328], other benign [OB; N=63], or other malignant [OM; N=60] adrenal masses.

	ACA Tumour diameter <4cm USM				OB Tumour diameter <4cm USM				OM Tumour diameter <4cm USM			
**Unenhanced CT >20HU or heterogeneity**	Low Risk	Moderate Risk	High Risk	Total	Low Risk	Moderate Risk	High Risk	Total	Low Risk	Moderate Risk	High Risk	Total
Negative	592	315	62	969	5	5	1	11	0	0	0	0
Positive	82	47	12	141	4	13	0	17	17	19	5	41
Total	674	362	74	1110	9	18	1	28	17	19	5	41

	ACA Tumour diameter ≥4cm USM				OB Tumour diameter ≥4cm USM				OM Tumour diameter ≥4cm USM			
**Unenhanced CT >20HU or heterogeneity**	Low Risk	Moderate Risk	High Risk	Total	Low Risk	Moderate Risk	High Risk	Total	Low Risk	Moderate Risk	High Risk	Total
Negative	102	45	12	159	11	9	1	21	1	0	0	1
Positive	26	19	14	59	5	9	0	14	10	8	0	18
Total	128	64	26	218	16	18	1	35	11	8	0	19

STARD Checklist

**TITLE OR ABSTRACT**			
	**1**	Identification as a study of diagnostic accuracy using at least one measure of accuracy (such as sensitivity, specificity, predictive values, or AUC)	1
**ABSTRACT**			
	**2**	Structured summary of study design, methods, results, and conclusions (for specific guidance, see STARD for Abstracts)	5
**INTRODUCTION**			
	**3**	Scientific and clinical background, including the intended use and clinical role of the index test	8
	**4**	Study objectives and hypotheses	9
**METHODS**			
Study design	**5**	Whether data collection was planned before the index test and reference standard were performed (prospective study) or after (retrospective study)	10
Participants	**6**	Eligibility criteria	10
	**7**	On what basis potentially eligible participants were identified (such as symptoms, results from previous tests, inclusion in registry)	10
	**8**	Where and when potentially eligible participants were identified (setting, location and dates)	10
	**9**	Whether participants formed a consecutive, random or convenience series	10
Test methods	**10a**	Index test, in sufficient detail to allow replication	11,12, Suppl.
	**10b**	Reference standard, in sufficient detail to allow replication	11,12
	**11**	Rationale for choosing the reference standard (if alternatives exist)	12
	**12a**	Definition of and rationale for test positivity cut-offs or result categories of the index test, distinguishing pre-specified from exploratory	11,12
	**12b**	Definition of and rationale for test positivity cut-offs or result categories of the reference standard, distinguishing pre-specified from exploratory	12
	**13a**	Whether clinical information and reference standard results were available to the performers/readers of the index test	11,12
	**13b**	Whether clinical information and index test results were available to the assessors of the reference standard	11,12
Analysis	**14**	Methods for estimating or comparing measures of diagnostic accuracy	12, Suppl.
	**15**	How indeterminate index test or reference standard results were handled	11,12,Suppl
	**16**	How missing data on the index test and reference standard were handled	Suppl.
	**17**	Any analyses of variability in diagnostic accuracy, distinguishing pre-specified from exploratory	Suppl.
	**18**	Intended sample size and how it was determined	12
**RESULTS**			
Participants	**19**	Flow of participants, using a diagram	28 (Fig 1)
	**20**	Baseline demographic and clinical characteristics of participants	14
	**21a**	Distribution of severity of disease in those with the target condition	14
	**21b**	Distribution of alternative diagnoses in those without the target condition	14
	**22**	Time interval and any clinical interventions between index test and reference standard	Suppl.
Test results	**23**	Cross tabulation of the index test results (or their distribution) by the results of the reference standard	24,25,29, 30, Suppl.
	**24**	Estimates of diagnostic accuracy and their precision (such as 95% confidence intervals)	15,16,17
	**25**	Any adverse events from performing the index test or the reference standard	N/A
**DISCUSSION**			
	**26**	Study limitations, including sources of potential bias, statistical uncertainty, and generalisability	20

	**27**	Implications for practice, including the intended use and clinical role of the index test	19,20,21
**OTHER INFORMATION**			
	**28**	Registration number and name of registry	10
	**29**	Where the full study protocol can be accessed	N/A
	**30**	Sources of funding and other support; role of funders	13

Supplementary References

1. Arlt W, Biehl M, Taylor AE, et al. Urine steroid metabolomics as a biomarker tool for detecting malignancy in adrenal tumors. *J Clin Endocrinol Metab* 2011; **96**(12): 3775-84.
2. WHO Classification of Tumours of Endocrine Organs. 4th ed: International Agency for Research on Cancer (IARC); Lyon, France; 2017.
3. Giordano TJ, Berney D, de Krijger RR, et al. Carcinoma of the Adrenal Cortex Histopathology Reporting Guide. International Collaboration on Cancer Reporting. Sydney, Australia; 2019.
4. Lenders JW, Duh QY, Eisenhofer G, et al. Pheochromocytoma and paraganglioma: an endocrine society clinical practice guideline. *J Clin Endocrinol Metab* 2014; **99**(6): 1915-42.
5. Nieman LK, Biller BM, Findling JW, et al. The diagnosis of Cushing's syndrome: an Endocrine Society Clinical Practice Guideline. *J Clin Endocrinol Metab* 2008; **93**(5): 1526-40.
6. Funder JW, Carey RM, Fardella C, et al. Case detection, diagnosis, and treatment of patients with primary aldosteronism: an endocrine society clinical practice guideline. *J Clin Endocrinol Metab* 2008; **93**(9): 3266-81.
7. Fassnacht M, Arlt W, Bancos I, et al. Management of adrenal incidentalomas: European Society of Endocrinology Clinical Practice Guideline in collaboration with the European Network for the Study of Adrenal Tumors. *Eur J Endocrinol* 2016; **175**(2): G1-G34.
8. Kohonen T. Self-Organizing Maps. 2nd ed. Berlin: Springer; 1997.
9. Biehl M. A no-nonsense GMLVQ demo code (Version 2.3). http://www.cs.rug.nl/~biehl/gmlvq.
10. Biehl M, Schneider P, Smith DJ, et al. Matrix relevance LVQ in steroid metabolomics based classification of adrenal tumors. European Symposium on Artificial Neural Networks, Computational Intelligence and Machine Learning
2012 25-27 April 2012 Bruges (Belgium); 2012.
11. Schneider P, Biehl M, Hammer B. Adaptive relevance matrices in learning vector quantization. *Neural Comput* 2009; **21**(12): 3532-61.
12. Biehl M, Hammer B, Villmann T. Prototype-based models in machine learning. *Wiley Interdiscip Rev Cogn Sci* 2016; **7**(2): 92-111.
13. Fawcett T. An introduction to ROC analysis. *Pattern Recogn Lett* 2006; **27**(8): 861-74.
